# Identifying reproducible resting state networks and functional connectivity alterations following chronic restraint stress in anaesthetized rats

**DOI:** 10.3389/fnins.2023.1151525

**Published:** 2023-05-22

**Authors:** Twain Dai, Bhedita J. Seewoo, Lauren A. Hennessy, Samuel J. Bolland, Tim Rosenow, Jennifer Rodger

**Affiliations:** ^1^School of Biological Sciences, University of Western Australia, Perth, WA, Australia; ^2^Perron Institute for Neurological and Translational Science, University of Western Australia, Perth, WA, Australia; ^3^Minderoo Foundation, Perth, WA, Australia; ^4^Centre for Microscopy, Characterisation and Analysis, Research Infrastructure Centres, University of Western Australia, Perth, WA, Australia

**Keywords:** functional connectivity, anxiety, magnetic resonance imaging, rat brain, animal model of depression

## Abstract

**Background:**

Resting-state functional MRI (rs-fMRI) in rodent models have the potential to bridge invasive experiments and observational human studies, increasing our understanding of functional alterations in the brains of patients with depression. A major limitation in current rodent rs-fMRI studies is that there has been no consensus on healthy baseline resting-state networks (RSNs) that are reproducible in rodents. Therefore, the present study aimed to construct reproducible RSNs in a large dataset of healthy rats and then evaluate functional connectivity changes within and between these RSNs following a chronic restraint stress (CRS) model within the same animals.

**Methods:**

A combined MRI dataset of 109 Sprague Dawley rats at baseline and after two weeks of CRS, collected during four separate experiments conducted by our lab in 2019 and 2020, was re-analysed. The mICA and gRAICAR toolbox were first applied to detect optimal and reproducible ICA components and then a hierarchical clustering algorithm (FSLNets) was applied to construct reproducible RSNs. Ridge-regularized partial correlation (FSLNets) was used to evaluate the changes in the direct connection between and within identified networks in the same animals following CRS.

**Results:**

Four large-scale networks in anesthetised rats were identified: the DMN-like, spatial attention-limbic, corpus striatum, and autonomic network, which are homologous across species. CRS decreased the anticorrelation between DMN-like and autonomic network. CRS decreased the correlation between amygdala and a functional complex (nucleus accumbens and ventral pallidum) in the right hemisphere within the corpus striatum network. However, a high individual variability in the functional connectivity before and after CRS within RSNs was observed.

**Conclusion:**

The functional connectivity changes detected in rodents following CRS differ from reported functional connectivity alterations in patients with depression. A simple interpretation of this difference is that the rodent response to CRS does not reflect the complexity of depression as it is experienced by humans. Nonetheless, the high inter-subject variability of functional connectivity within networks suggests that rats demonstrate different neural phenotypes, like humans. Therefore, future efforts in classifying neural phenotypes in rodents might improve the sensitivity and translational impact of models used to address aetiology and treatment of psychiatric conditions including depression.

## 1. Introduction

Depression is a leading cause of global disability and a highly heterogenous disorder, characterized by affective and cognitive symptoms. Accumulating studies have suggested that these core symptoms of depression are associated with disrupted affective and cognitive brain networks, revealed by magnetic resonance imaging (MRI) and computational approaches that can map the brain connectome non-invasively (Fornito et al., 2015). Resting-state functional MRI (rs-fMRI) is a non-invasive and powerful MRI modality commonly applied to investigate resting-state functional organization in the brain at the macroscale level (Xu et al., 2022) through detecting the spontaneous fluctuations of the blood oxygen level dependent (BOLD) signal (Ogawa et al., 1990). The resting-state functional organization is generally referred to as resting-state network (RSN), such as the default mode network (DMN), central executive network (CEN), and salience network (Paulus and Stein, 2010; Williams, 2017). Several consistent functional connectivity changes within and between RSNs have been observed in rs-fMRI studies of depression in humans (Mulders et al., 2015). For instance, increased connectivity is presented within the anterior DMN, as well as between the anterior DMN and salience network. In contrast, decreased connectivity is shown between the posterior DMN and CEN.

RSNs are not unique to humans, some homologous RSNs have also been observed in healthy rodents using rs-fMRI (Xu et al., 2022; Grandjean et al., 2023). For example, the core brain structures of DMN in humans, such as the cingulate, retrosplenial, and prefrontal cortex, are identified to form DMN-like functional organization in rats and mice (Lu et al., 2012; Hsu et al., 2016; Mandino et al., 2022). Animal models have traditionally been a useful tool in the study of aetiology and treatment of depression due to the ethical and practical limitations associated with controlling the natural development of a disease and dissecting the neurobiological mechanism in humans (Herzog et al., 2018; Pais-Roldan et al., 2021). The relevance of animal depression models is often controversial because no single rat model can perfectly replicate all aspects of clinical features of depression, such as depressed mood and suicidal thoughts (Harro, 2019). However, a perfect rat model of depression that exhibits all the clinical features of depression-relevant behaviors is arguably unnecessary because even patients usually do not manifest every aspect of diagnostic criteria of depression (Krishnan and Nestler, 2008). Therefore, it would still be beneficial to investigate and compare the functional connectivity between and within RSNs in health and subsequent depression models within the same animals.

To date, limited rs-fMRI studies in animal models of depression have investigated functional alterations within and between RSNs in response to interventions that induce stress and anxiety (Henckens et al., 2015; Grandjean et al., 2016; Nephew et al., 2018; Seewoo et al., 2020; Hennessy et al., 2022). Majority of these rodent studies have applied chronic restraint stress (CRS), which is a popular, simple, and validated depression model. It has been shown to induce changes in behaviors, gene expression, and protein, which are similar to those in patients with depression (Becker et al., 2021). Each of these animal studies has identified different aspects of dysfunctional RSNs that are claimed to be comparable to humans with depression. However, there is no consistent findings within these rodent rs-fMRI studies. Several reasons may explain the discrepant results, including limited sample sizes, different methodologies applied to construct and analyse RSNs, and different protocols used to induce depression-like behaviors and neurological alterations.

A major limitation in current rodent rs-fMRI studies is that there has been no consensus on healthy baseline RSNs that are reproducible in rodents (Becerra et al., 2011) regardless growing efforts has been devoted to studying RSNs in healthy animals. Identifying reproducible RSNs at baseline or in healthy condition is crucial for investigating alterations of functional connectivity following interventions used to model depression in animals. To date, limited studies have attempted to construct reproducible RSNs in rodents and they also suffer from a major pitfall of limited sample sizes (Becerra et al., 2011). Moreover, no study has examined the effects of depression models on the alterations of functional connectivity following a construction of reproducible RSNs. Therefore, the present study first aims to construct reproducible RSNs in a large dataset of 109 healthy rats and then evaluate functional connectivity changes within and between these RSNs following a depression model in the same animals.

## 2. Materials and methods

### 2.1. Ethics statement

All experimental procedures adhered to the ethics guideline of the University of Western Australia Animal Ethics Committee (RA/3/100/1640) and the National Health and Medical Research Council’s Australian code for the care and use of animals for scientific purposes. All investigators had obtained the Permission to Work with Animals and were trained by the UWA Program in Animal Welfare, Ethics, and Science.

### 2.2. Rodent MRI data

#### 2.2.1. Animals

The MRI data analysed in the present study was a combined dataset of rodent cohorts from previous experiments conducted by our lab in 2019 and 2020. Briefly, 109 male Sprague—Dawley rats (aged 6–7 weeks and weighing 150–250 g on arrival; *N* = 12 from Seewoo et al. (2020), *N* = 56 from Hennessy et al. (2022) and *N* = 41 unpublished) were sourced from the Animal Resources Centre (Canning Vale, WA). All rats were housed in pairs under a standard 12-h light–dark cycle with *ad libitum* food and water, in a temperature-controlled and spacious laboratory room located at UWA’s Animal Care Unit, M block building (Nedlands, WA). All animals were habituated to the new environment for one week after their arrival and prior to experiments.

96 rats underwent CRS for 2.5 h per day for 13 consecutive days. These animals’ MRI scans were conducted at baseline (one or two days before the first CRS procedure) and post-CRS (two or three days after the final CRS procedure). The remaining animals (*N* = 13), served as controls: they did not undergo CRS but received MRI imaging twice at the same interval applied to the intervention group.

#### 2.2.2. Chronic restraint stress protocols

CRS was performed on a bench situated in one corner of the laboratory room, with rats placed in a transparent plastic tube facing the wall to mitigate visual distractions. Each session started between 12:30 and 13:00 pm, lasting for 2.5 h daily to reduce the influence of circadian rhythm. Restraint tubes with adjustable tail gates used to restrict the free movement of animals were adjusted to match their body mass and size throughout 13 days of CRS (Seewoo et al., 2020). During CRS sessions, rats in the control group stayed in their home cages. Following CRS, rats in the intervention group were returned to their home cages.

#### 2.2.3. MRI anaesthetic protocol

Animals were transferred to the National Imaging Facility located in the Harry Perkins Institute of Medical Research (Nedlands, WA) for MRI scan on the assigned day. The rat was weighed and then pre-anaesthetised in an induction box with 4% isoflurane in medical air (2 L/min). Once fully anaesthetised, the animal was transferred to a heated imaging bed and anaesthesia was initially administered through a nose cone with 4% isoflurane in medical air (1 L/min). The animal’s vital status was monitored using a PC-SAM Small Animal Monitor (SA Instruments Inc., 1,030 system). Once the respiratory rate dropped to 55–60 breaths/min, isoflurane concentration was adjusted to 2% in medical air (1 L/min). After the animal was stabilized on 2% isoflurane for at least 2 min, medetomidine was delivered subcutaneously, with an initial 0.05–0.1 mg/kg bolus injection and continuous 0.15 mg/kg/h infusion. Meanwhile, the isoflurane concentration was gradually reduced to 0.5–0.75% based on the animal’s respiratory rate (Seewoo et al., 2020). The combined use of isoflurane and medetomidine can induce similar functional connectivity within RSNs, compared to animals in an awaken condition (Paasonen et al., 2018). Moreover, this anaesthetic protocol can maintain strong cortical–cortical and cortical–subcortical connectivity in animals (Grandjean et al., 2014; Bukhari et al., 2017). To evaluate the potential influence of time elapsed following the induction of medetomidine on RSNs, 14 imaging sessions were randomly selected, in which the time elapsed was plotted against network metrics (see methods 2.3.5). No correlation (data not shown; *r* < 0.001) was identified and the average time elapsed was 32 min (*SD* = 4). After the imaging session, animals were administered a 0.15 mg/kg injection of atipamezole to antagonize medetomidine. Periodical monitoring for adverse events after MRI scan was performed in the following 24 h.

#### 2.2.4. MRI acquisition

Rats were scanned using a 9.4 T Bruker Biospec 94/30 pre-clinical MRI scanner (Bruker BioSpin GmbH, Germany) with a BGA-12SHP imaging gradient system, a 72 mm or 86 mm volume resonator transmit coil (depending on hardware availability), a rat brain surface quadrature receive coil, and Avance III console. ParaVision 6.0.1 software of the Bruker controlled scanning sequences and operation for structural MRI and rs-fMRI acquisition (Seewoo et al., 2018; Han et al., 2019). High-resolution T2-weighted anatomical images with 21 coronal slices were acquired using an accelerated multi-slice 2D rapid acquisition with relaxation enhancement (RARE) sequence. A single-shot gradient-echo echo planar imaging (EPI) sequence was applied to acquire resting-state functional images with 21 coronal slices. MRI scanning parameters were summarized in Table 1. Both raw images for each scan session were compiled in one Para Vision 6.0.1 package in the format of PvDatasets. A total of 218 MRI packages were acquired at two time points (baseline and post-CRS) and included in the following data processing and analysis workflow.

**Table 1:** MRI scanning protocols for structural MRI and rs-fMRI acquisition.

Parameters	Structural MRI (RARE)	rs-fMRI (EPI)
Repetition time	2.5 s	1.5 s
Echo time	33 ms	11 ms
Scan time	2 min 55 s	7 min 30 s
Matrix size	280 × 280	94 × 70
Field of view (FOV)	28 × 28 mm^2^	28.2 × 21 mm^2^
Spatial resolution	0.1 mm × 0.1 mm	0.3 mm × 0.3 mm
Slice thickness	1 mm	1 mm
Slice gap	0.05 mm	0.05 mm
Slice	21	21
Repetition	1	300
Receiver bandwidth	34722 Hz	300000 Hz
Acceleration factor	8	–
Order automatic ghost correction	**–**	1
Fat suppression	Yes	Yes
B0 shimming	No	Yes
Read orientation	Left to right	Left to right

### 2.3. MRI data processing and analysis

The workflow was remotely operated on the MASSIVE’s super-computing desktop (Goscinski et al., 2014) and mostly consisted of common processing steps (Moher Alsady et al., 2016; Bajic et al., 2017; Seewoo et al., 2021). Detailed scripts for executing the series of steps can be found in Supplementary material S1. Most of the processing and analysis steps were performed with the Functional MRI of the Brain (FMRIB) Software Library 6.0.3 (FSL 6.0.3; Jenkinson et al., 2012), unless otherwise specified.

#### 2.3.1. Common image pre-processing

The pre-processing for each data package was batch processed as follows: (1). Extract DICOM (Bidgood et al., 1997) of both raw images from PVDatasets packages; (2). Convert DICOM into NifTi using dcm2niix converter (version: 6-October-2021 for Linux system; Li et al., 2016); (3). Reorient images in the radiological view (left-anterior-superior axes); (4). Correct bias field signals for anatomical images using 3D Slicer (version: 4.8.1; Fedorov et al., 2012); (5). Strip the skull for anatomical images and create an individualized brain mask for the next step; (6). Extract the brain for functional images using the brain mask; (7). Upscale the voxel size of functional images by a factor of 10 (Bajic et al., 2017). Additionally, the quality of reorientation and brain extraction were visually inspected using FSL/slices. Only one anatomical and one functional package failed to reorient within the batch processing and were re-processed separately to correct their orientation.

#### 2.3.2. Further functional image pre-processing

Upscaled functional brain images were further pre-processed on the FSL/MELODIC interface with registration, pre-statistic processing, and single-session independent component analysis (ICA). To elaborate, upscaled functional brain images were first registered to their corresponding upscaled anatomical brain images and normalized to a Sprague–Dawley rat brain atlas, which was down sampled by a factor of eight from the Waxholm Space atlas (RRID:SCR_017124; Papp et al., 2014). The down-sampled atlas (voxel size: 3.125 × 3.125 × 3.125 mm^3^) was used to better match the voxel size of functional data and all subsequent processing was performed in this atlas space. Following registration, pre-statistics processing was applied with motion correction (Jenkinson et al., 2002), a temporal high pass filter cut-off of 100 s. No spatial smoothing was applied at this stage (Moher Alsady et al., 2016; Bajic et al., 2017). Single-session ICA was conducted using Probabilistic ICA (Beckmann and Smith, 2004). Finally, outputs of single-session ICA in native space were further de-noised applying FMRIB’s ICA-based Xnoiseifier (FIX; version: 1.068; Griffanti et al., 2014; Salimi-Khorshidi et al., 2014) with a trained network (trained-weights file) that can distinguish noise and signals, at a threshold of 20. The trained network was generated with FIX using 50 sets of hand-labelled single-session ICA components, based on each component’s spatial maps, frequency, and time-course (Salimi-Khorshidi et al., 2014; Seewoo et al., 2021). Global signal regression was not used to denoise in the present study due to its potential pitfall of introducing spurious anti-correlations (Murphy et al., 2009). Finally, de-noised functional brain images were registered to the down-sampled atlas to construct de-noised and registered functional images.

#### 2.3.3. Group-level ICA

Instead of whole brain, the multi-subject temporal concatenation group-ICA was applied on a large region of interest (ROI) comprising cerebral cortex, hippocampus, amygdala, thalamus, basil ganglia, claustrum, and hypothalamus and colliculi. Most of these structures are considered components of RSNs in humans and animals, while the colliculi and piriform cortex are typically found in rodents as additional components (Jonckers et al., 2011; Seitzman et al., 2019; Smith et al., 2019).

##### 2.3.3.1. Atlas mask generation for the ROI

The ROI atlas mask was generated as follows: (1). Extract high-resolution masks for substructures of the ROI based on their label ID from the Waxholm Space atlas (RRID:SCR_017124; Papp et al., 2014) using ITK-SNAP/Convert3D (version: 1.0.0; Yushkevich et al., 2006); (2). Resample high-resolution masks of each substructure to the down-sampled atlas (see section 2.3.2) to create low-resolution masks and combine these low-resolution masks to form the final ROI mask (See Supplementary material 1 3.1 for detailed scripts, Supplementary material 2 for ROI and substructure masks, and Supplementary material 3 for the volume of each substructure).

##### 2.3.3.2. Spatial smoothing effect on optimal group-ICA dimensionality

An optimal group-ICA dimensionality was estimated using the mICA toolbox, which estimates correlation values using random split-half sampling or test–retest analyses for a range of dimensionalities (Moher Alsady et al., 2016). Briefly, all de-noised and normalized functional images (see section 2.3.2) acquired at baseline (*N* = 109) and the ROI atlas mask were imported to the toolbox. The rationale for excluding rs-MRI data acquired at post-CRS is to avoid CRS-related resting-state alteration in identifying ICA template and to increase the sensitivity of further data processing in detecting group differences following CRS (Seewoo et al., 2021). Random split-half sampling with 50 repetitions were performed at 20 different levels of dimensionality ranging from 10 to 200 components with an interval of 10. For each repetition, MELODIC group-ICA was carried out on both split-half groups (*N* = 54 samples/group) and a cross-correlation matrix between components’ spatial maps was calculated using Pearson’s correlation. Hungarian sorting algorithm (Kuhn, 2005) was applied to match intergroup components and maximize the summed correlation of all component pairs. Mean correlation and 95% confidence interval (CI) over 50 repetitions were calculated and used to estimate the optimal dimensionality. Whether group-ICA in each dimensionality failed to converge components was also monitored. This analysis was repeated for four different Gaussian kernels of full-width half maximum (FWHM) at 6.25, 9.375, 12.5, and 15.625 mm (corresponding to twice, threefold, fourfold, fivefold the atlas voxel size; Mikl et al., 2008; Chen and Calhoun, 2018). The optimal group-ICA dimensionality under four Gaussian kernels was determined based on the global maximum of correlation outputs (Moher Alsady et al., 2016). Resultant correlation outputs of 80 combinations (20 levels of ICA dimensionality with four Gaussian kernels) were presented in a curve plot. A combination with the maximum value, corresponding to the global maximum of correlation value presented in the curve plot, was considered as an optimal dimensionality. Group-ICA outputs of all split-half sampling groups (*N* = 100) at the resultant dimensionality and Gaussian kernel were ready for the following processing.

##### 2.3.3.3. Ranking and averaging independent component analysis by reproducibility

Ranking and averaging independent component analysis by reproducibility (RAICAR) ranks and selects components based on the reproducibility over repeated ICA realizations, in which a cross-realization correlation matrix is constructed to align components (Yang et al., 2008). Each aligned component over multiple realizations is averaged to generate the final spatial maps of that component. RAICAR is a promising tool to identify robust reproducible components to construct reproducible RSNs following an estimation of optimal decomposition dimensionality for ICA approaches. Rather than performing one run of group-ICA at the optimal dimensionality and Gaussian kernel on all baseline data, the present study applied the 100 group-ICA maps from the previous step (see section 2.3.3.2) to gRAICAR toolbox (Yang et al., 2012) in MATLAB (version: r2019b). As running group-ICA on the same dataset several times does not produce the same spatial map and there is between-subject variability (run-to-run variability) in ICA results, conducting a RAICAR based on multiple group-ICA maps would expect to generate ICA components that are fair representatives of RSNs and being reproducible across multiple runs and subjects (Becerra et al., 2011; Pendse et al., 2011). Therefore, although gRAICAR was originally performed on individual subjects’ ICA maps or functional images, this study implemented the toolbox to align and rank components over the 100 group-ICA maps. An averaged spatial map and intergroup variability (similarity and confidence of contribution) for each aligned and ranked component/node was generated. Nodes were labelled based on intergroup variability in an ascending manner, with node one having the lowest variability. The mean inter-group similarity and ratio of significant groups contributing to each node were reported with column plots. The significant of a group was set as more than 0.05 confidence of the group load [detailed explanation see Yang et al. (2012)]. Additionally, results of group load index and confidence of group load for each component were plotted and compiled in Supplementary material 4.

Spatial maps of all resultant nodes were then merged using FSL/fslmerge command and parcellated using mixture modelling approach based on the thresholded z-transformed results of each node (Woolrich et al., 2005; Moher Alsady et al., 2016). A numerical label is assigned to each voxel based on the node with the highest Z-value at that voxel. As a result, each resultant node’s spatial boundary was determined. All the nodes were merged to generate a group-ICA template for a network analysis.

#### 2.3.4. Network modelling

The group-ICA template for the ROI was mapped onto baseline data (*N* = 109; de-noised and normalized functional images, spatially smoothed at resultant Gaussian kernel in section 2.3.3.2) to derive subject-specific time series for all nodes using FSL/dual regression (Nickerson et al., 2017). Images of nodes were created using FSL/slices summary. These time series and images of nodes were then fed into FSLNets (v0.6) in MATLAB (version: r2019b) to perform network modelling. A group average network hierarchy of these nodes was generated based on full correlation using Ward’s method. Clusters of highly correlated nodes were merged into large-scale functional networks. For each template network, brain structures were then identified and reported. Absolute volume of each structure with its average Z-score (representing levels of resting-state activity) were extracted using FSL/fslstats and percentage volume against its anatomical total (representing the relative size or spatial extent of resting-state activity) was then calculated (Supplementary material 3). These metrics or spatial characteristics were used to classify networks (Becerra et al., 2011). Additionally, the volumes with its average *Z*-score of brain structure covered by each node within each network were extracted and summarized in Supplementary material 5.

#### 2.3.5. Comparison of CRS and control groups to baseline

To compare the differences in network connectivity for the CRS group between baseline and post-CRS, as well as for the control group between baseline and the second scan session, statistical analysis was conducted in parallel. Due to the difference in the sample size (13 in control, 96 in CRS group), the CRS group was not compared directly with the control group. However, by analysing control and CRS group in parallel, this longitudinal (repeated measures) approach increases the statistical power as each animal was compared to itself, meaning participant/animal variables are controlled at both time points. It also adds biological relevance because it allows investigation of individual susceptibility and response, information which is relevant to future precision medicine approaches (see discussion). In an exploratory statistical analysis, the control (*N* = 13) and CRS (*N* = 96) groups were compared to each other directly at the second time point. But the very different sample size (along with uncontrolled animal variables) resulted in low effect sizes and thus direct comparison approach was not deemed suitable (data not shown).

##### 2.3.5.1. Identification of differences between networks

For the intervention group, the classified networks were merged using FSL/fslmerge and then mapped onto all data acquired at baseline and post-CRS (*N* = 192; de-noised and normalized functional images, spatially smoothed at resultant Gaussian kernel in section 2.3.3.2) to derive subject-specific time series for all networks using FSL/dual regression (Nickerson et al., 2017). These time series were then fed into FSLNets (v0.6) in MATLAB (version: r2019b) to determine if there were differences in the direct connection between networks following CRS. Fisher’s r-to-Z transformation (Smith et al., 2011) and ridge-regularized partial correlation was applied to improve the mathematical robustness and achieve better estimation (Smith et al., 2013). The edge [direct connection; definition see Menon and Krishnamurthy (2019)] strength between each pair of networks were compared at baseline and post-CRS using paired permutation t-test with randomize (5,000 permutations, familywise error rate corrected for multiple comparisons across all edges). Raw values of significant edges (*p* < 0.05) were extracted from MATLAB and imported to RStudio (version: 2021.09.2 + 382) to estimate the effect size using ‘dabestr’ (Ho et al., 2019). For the control group, the same methods described above were applied to all data (*N* = 26; de-noised and normalized functional images, spatially smoothed at resultant Gaussian kernel in section 2.3.3.2). Results showing significant differences (*p* < 0.05) were reported with network images, estimation plots, mean ± SD, permutation *p* values, and Cohen’s d with 95% CI. Raw values of significant edges between networks can be found in Supplementary material 6.

##### 2.3.5.2. Identification of differences within each network

For the intervention and control group, the same methods described in section 2.3.5.1 were applied to each resultant network template from section 2.3.4 to detect if there were differences in the direct connection between nodes within each network at baseline and post-CRS, respectively. Results showing significant differences (*p* < 0.05) within each network were reported with node images, estimation plots, mean ± SD, permutation p values, Cohen’s d with 95% CI for the intervention group, and Hedges’ g with 95% CI for the control group. Raw values of significant edges within each network can be found in Supplementary material 6.

##### 2.3.5.3. Inter-subject variability of edge strength in the intervention group

Raw values (edge value of individual animals) of abovementioned significant edges between and within networks were grouped into three categories at baseline and post-CRS: positive (above 0.2), none (between −0.2 and 0.2), negative (below −0.2) partial correlation (Hevey, 2018). Changes in the edge strength following CRS were classified into 11 categories, including positive to more positive, positive to less positive, positive to none, positive to negative, negative to more negative, negative to less negative, negative to none, negative to positive, none to none, none to positive, none to negative. The distribution of edge changes of individual animals falling within these categories were used to indicate if there was high individual variability in terms of these edges showing significance at group level identified in sections 2.3.5.1 and 2.3.5.2. A summary of animal counts of edges showing positive, negative, and no partial correlation for each significant edge at both time points, and changes in the edge strength following CRS can be found in Supplementary material 6.

## 3. Results

### 3.1. Spatial smoothing effect on optimal group-ICA dimensionality

**Figure 1:**
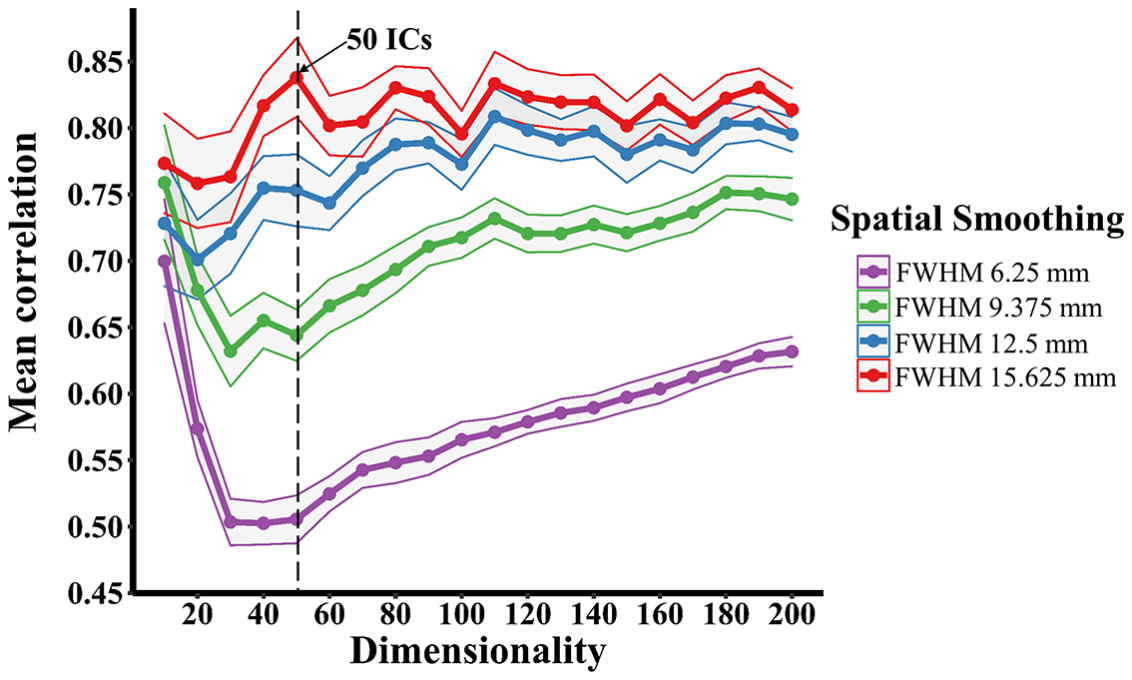
**Spatial smoothing effects on determining the optimal group-ICA dimensionality**. The dimensionality-reproducibility curves under four different Gaussian kernels showed that an increase in the spatial smoothing resulted in an overall increase in the correlation of group-ICA spatial maps. The number of components used in the further group-ICA decomposition was derived from the curves, where 50 independent components (ICs; arrow) was the global maximum. The Curves were plotted with mean ± 95% CI.

There was no failure of convergence in the group-ICA across all dimensionality levels under four different Gaussian kernels. As shown in Figure 1, an increase in the spatial smoothing resulted in an increase in the correlation of group-ICA spatial maps at each dimensionality ranging from 20 to 200. But there were similarly strong correlations (ranging from 0.70 to 0.77) at a dimensionality of 10 regardless of spatial smoothing. Under Gaussian kernels of FWHM 6.25 and 9.375 mm, the correlation value plummeted at first and increased gradually from the dimensionality of 50. In contrast, correlation values stabilised above 0.7, demonstrating strong or very strong associations across all dimensionality levels under Gaussian kernels of FWHM 12.5 and 15.625 mm. The correlation value (mean: 0.84, 95% CI: 0.03) at the dimensionality of 50 and Gaussian kernels of FWHM 15.625 mm was highest amongst all and considered the optimal dimensionality for further analysis.

### 3.2. RAICAR

**Figure 2:**
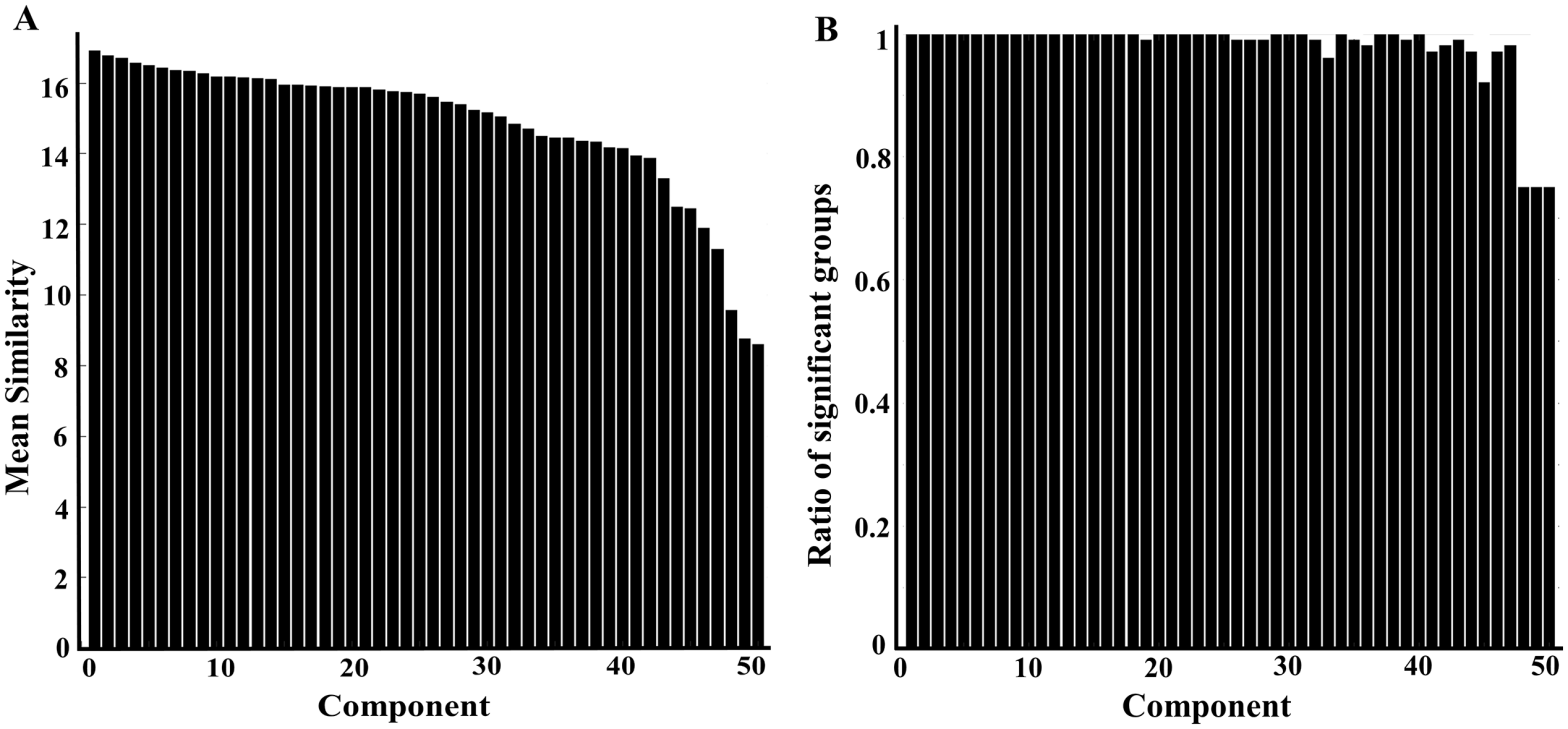
**Column charts showing intergroup variability at the dimensionality of 50 following RAICAR over 100 group-ICA spatial maps**. **(A)** Displaying the mean similarity of aligned 50 components. A higher value indicates the aligned component is more consistently found in different groups. **(B)** Displaying the proportion of groups contributed to the given aligned component. The first 47 components have a ratio of more than 0.95 (95/100 groups), indicating very strong intergroup consistency.

The resultant 50 components (optimal dimensionality) were aligned and ranked over 100 group-ICA spatial outputs with gRAICAR. Node 1 had the highest similarity and node 50 had the lowest similarity (Figure 2A). The gRAICAR also revealed that 47 nodes presented very strong inter-group consistency with a ratio of significant groups over 0.95 (Figure 2B). The last three components also had a ratio around 0.75 demonstrating strong inter-group consistency. Among these 50 reproducible components, seven were bilateral nodes, including node 4, 19, 23, 31, 37, 39, and 50 (substructures comprising these nodes see Supplementary material 5).

### 3.3. Networks

**Figure 3:**
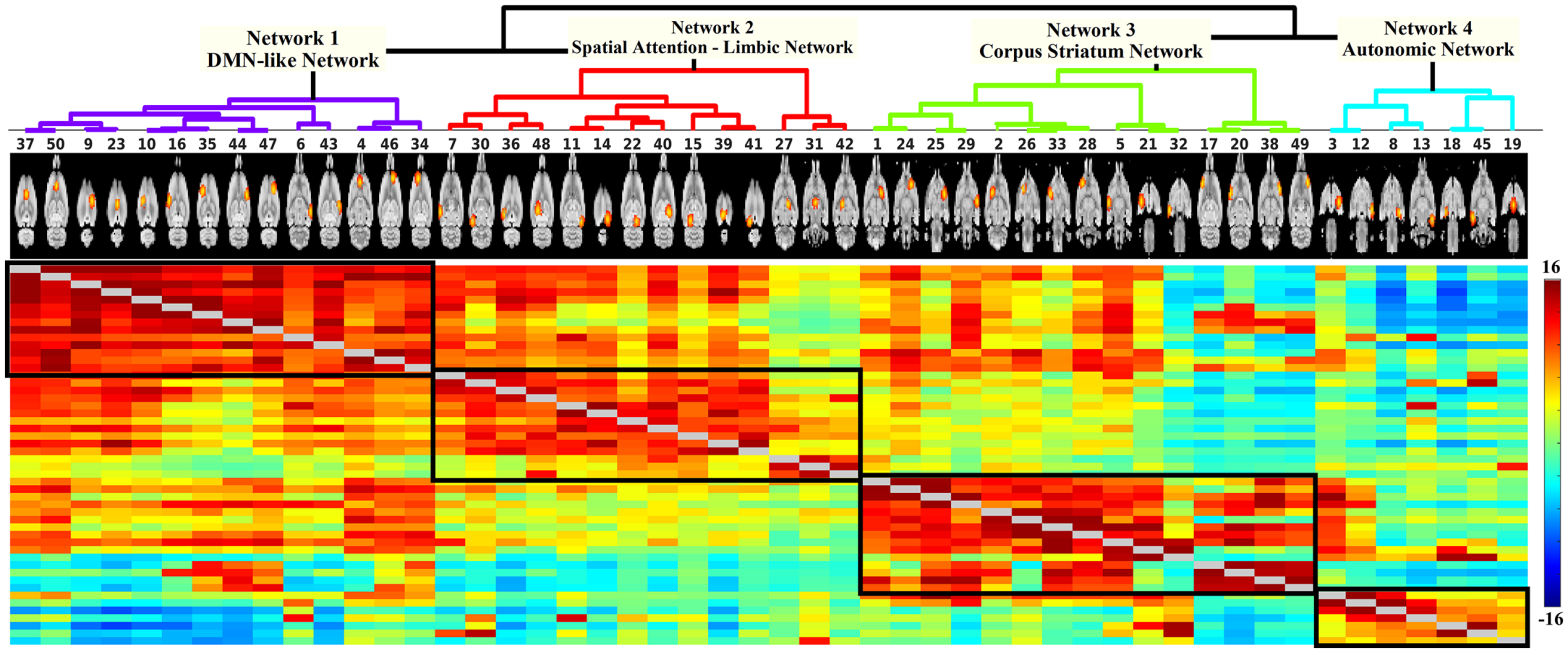
**Hierarchical network of 50 components**. Each node is denoted by one column. The coloured matrix demonstrates the full correlation (below and above the diagonal) of the time series between pairs of components. Darker red indicates a higher positive correlation, light green indicates no correlation, and darker blue represents higher negative correlation. The hierarchical analysis defined four major clusters (black box). Based on the spatial characteristics of each cluster, they were categorized into DMN-like network (14 nodes), spatial attention-limbic network (14 nodes), corpus striatum network (15 nodes), and autonomic network (7 nodes).

**Figure 4:**
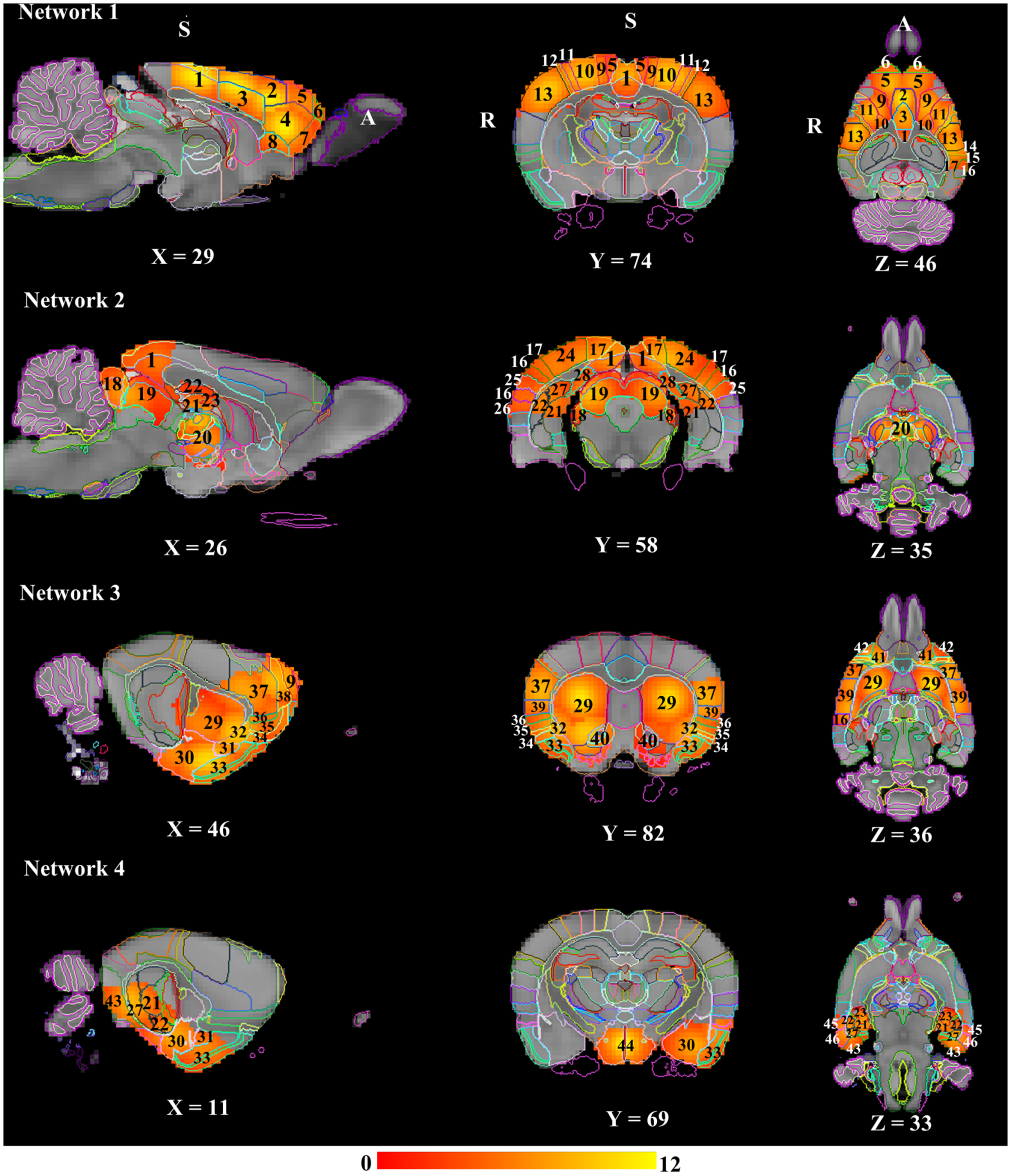
**Resting state networks in anaesthetised rats**. All the networks presented a marked bilateral organisation. These spatial maps were overlayed on the down-sampled atlas and labelled based on the Waxholm Space atlas (RRID: SCR_017124; Papp et al., 2014). Numbers denote: 1: Retrosplenial cortex; 2: Cg1; 3: Cg2; 4: Prelimbic cortex; 5: Secondary motor cortex; 6: Frontal association cortex; 7: Medial orbital cortex; 8: Infralimbic cortex; 9: Primary motor cortex; 10: Primary somatosensory area hindlimb representation; 11: Primary somatosensory area forelimb representation; 12: Primary somatosensory area dysgranular zone; 13: Primary somatosensory area barrel field; 14: Primary somatosensory area trunk representation; 15: Parietal association cortex; 16: Secondary auditory cortex; 17: Secondary visual cortex; 18: Inferior colliculus; 19: Superior colliculus; 20: Thalamus; 21: Dentate gyrus; 22: CA1; 23: CA3; 24: Primary visual cortex; 25: Primary auditory cortex; 26: Temporal association cortex; 27: Subiculum; 28: Presubiculum; 29: Caudate Putamen; 30: Amygdala; 31: Endopiriform nucleus; 32: Claustrum; 33: Piriform cortex; 34: Agranular insular cortex; 35: Dysgranular insular cortex; 36: Granular insular cortex; 37: Primary somatosensory area face representation; 38: Frontal association area 3; 39: Secondary somatosensory area; 40: Nucleus accumbens; 41: Lateral orbital cortex; 42: Dorsolateral orbital cortex; 43: Medial entorhinal cortex; 44: Hypothalamus; 45: Perirhinal cortex; 46: Lateral entorhinal cortex. A: Anterior; R: Right; S: Superior.

Network modelling based on hierarchical clustering detected four common patterns of functional connectivity in the rat brain. The data-driven approach merged the 50 nodes into four major clusters (Figure 3). Major structures covered by each network template are visualized in Figure 4 and volumes of structures with their average *Z*-score are summarized in Supplementary material 3. These networks are described below and listed in the left-to-right order of the hierarchical tree, including DMN-like network, spatial attention-limbic network, corpus striatum network, and autonomic network. Spatial symmetry was observed in some homologous brain regions within these networks.

#### 3.3.1. Network 1 – DMN-like network (14 nodes)

The network was predominantly cortical. Most of the DMN components (cingulate, anterior retrosplenial, prelimbic, orbital, infralimbic, and frontal association cortex) demonstrated bilateral symmetry in the resting-state activity. The spatial extent of resting-state activity in the cornu ammonis/CA, parietal association and visual cortex was larger in the left hemisphere, whereas the resting-state activity of temporal association, auditory, and perirhinal cortex was completely absent in the right hemisphere. Moreover, the resting-state activity of sensorimotor structures were mostly symmetrical in the network.

#### 3.3.2. Network 2 – spatial attention-limbic network (14 nodes)

The homologous brain regions presenting symmetrical resting-state activity were identified as follows: inferior colliculi, superior colliculi, posterior retrosplenial cortex, dorsal hippocampus, and thalamus. The spatial extent of resting-state activity of the visual, auditory, temporal association, postrhinal, and medial entorhinal cortex was larger in the right than left hemisphere, whilst parietal association, perirhinal, and primary somatosensory (trunk representation) cortex only had unilateral resting-state activity in the right hemisphere.

#### 3.3.3. Network 3 – corpus striatum network (15 nodes)

Brain substructures demonstrating bilateral symmetry in the resting-state activity included corpus striatum (nucleus accumbens, globus pallidus, ventral pallidum, and caudate putamen), claustrum, insular, orbital, motor, secondary somatosensory cortex. The spatial extent of resting-state activity of the endopiriform nucleus, perirhinal, piriform cortex was larger in the right than left hemisphere, whereas the resting-state activity of amygdala, CA, lateral entorhinal cortex was totally absent in the left hemisphere.

#### 3.3.4. Network 4 – autonomic network (7 nodes)

The network was predominantly subcortical. Bilateral symmetry in the resting-state activity was observed in the following subcortical structures: hypothalamus, ventral hippocampus, and substantia nigra. The spatial extent of resting-state activity of amygdala and adjacent cortical regions (lateral and medial entorhinal cortex) was larger in the left than right hemisphere, but the endopiriform nucleus and piriform cortex only demonstrated unilateral resting-state activity in the left hemisphere.

### 3.4. Changes in the functional connectivity between networks following CRS

**Figure 5:**
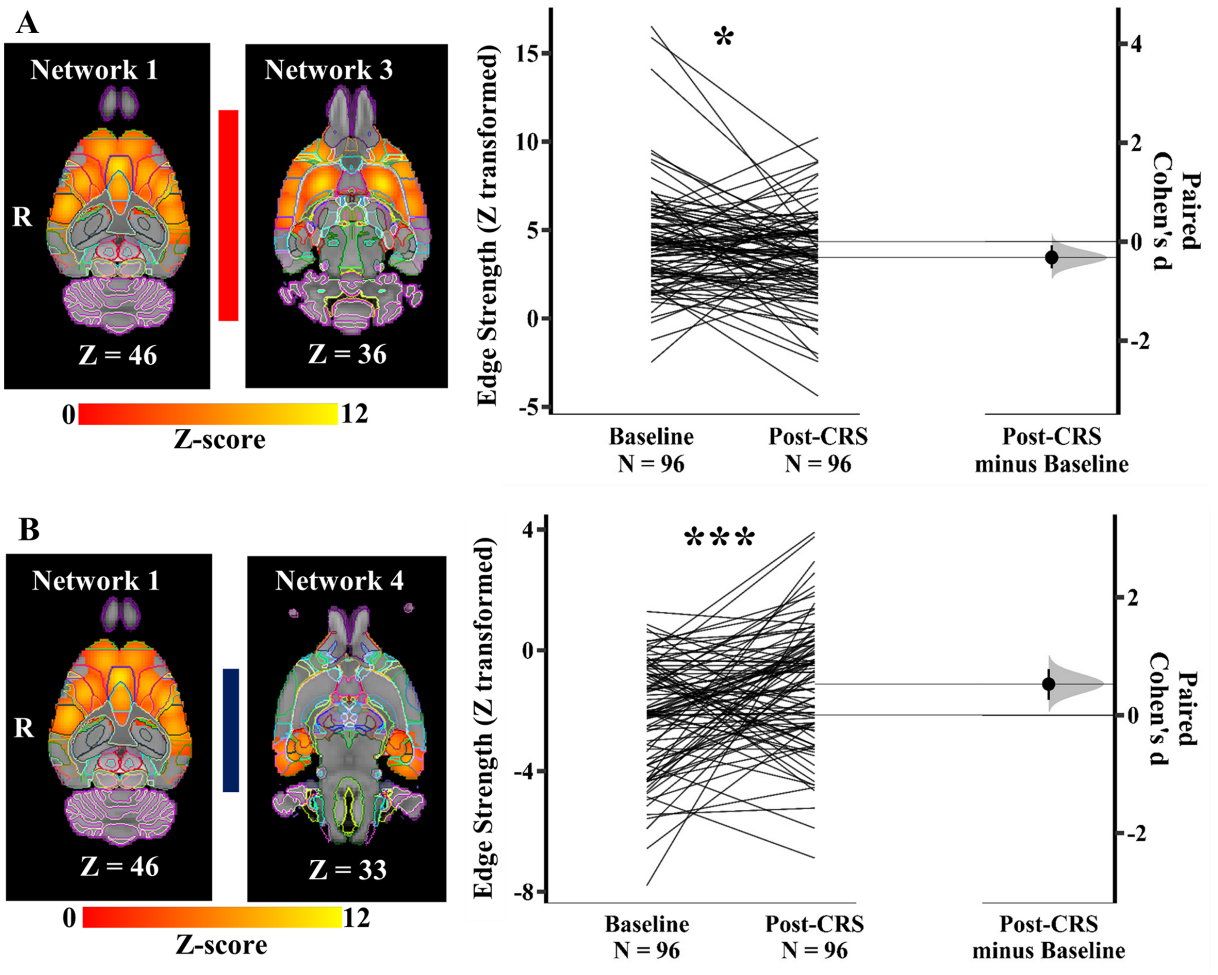
**Significant edge difference following CRS between networks**. **(A)** The connection strength between the DMN-like network (network 1) and corpus striatum network (network 3) displayed on the left showed a decrease following CRS. On the right, Gardner-Altman estimation plot showing a weak effect size (Cohen’s d: −0.32) in the edge strength between these two networks (* denotes *p* < 0.05). The raw data is plotted on the left axis as a slopegraph, with paired Cohen’s d plotted as a bootstrap sampling distribution on the right axis. Each paired set of observations is connected by a line. **(B)** The anticorrelation between the DMN-like network (network 1) and autonomic network (network 4) decreased significantly following CRS. The images of networks are displayed on the left. On the right, Gardner-Altman estimation plot showing a weak effect size (Cohen’s d: 0.53) in the edge strength between these two networks (*** denotes *p* < 0.001). The coloured bar joining each pair of networks indicates the overall group-average connection strength. Longer suggests a stronger connection, red indicates positive, and blue means negative or anticorrelated. R: Right.

As shown in Figure 5A, the edge/direct connection strength between the DMN-like network (network 1) and corpus striatum network (network 3) showed a decrease, but with weak effect size following CRS (baseline: 4.34 ± 3.02; post-CRS: 3.45 ± 2.56; *p* value: 0.044; Cohen’s d: −0.32; 95% CI: −0.54 ~ −0.07). In contrast, the connection strength between the DMN-like network (network 1) and autonomic network (network 4) increased significantly with a medium effect size following CRS (Figure 5B; baseline: −2.15 ± 1.84; post-CRS: −1.12 ± 2.06; *p* value: 0.0006; Cohen’s d: 0.53; 95% CI: 0.26 ~ 0.78). In other words, the anticorrelation between DMN-like network and autonomic network decreased significantly following CRS.

### 3.5. Changes in the functional connectivity within each network following CRS

**Figure 6:**
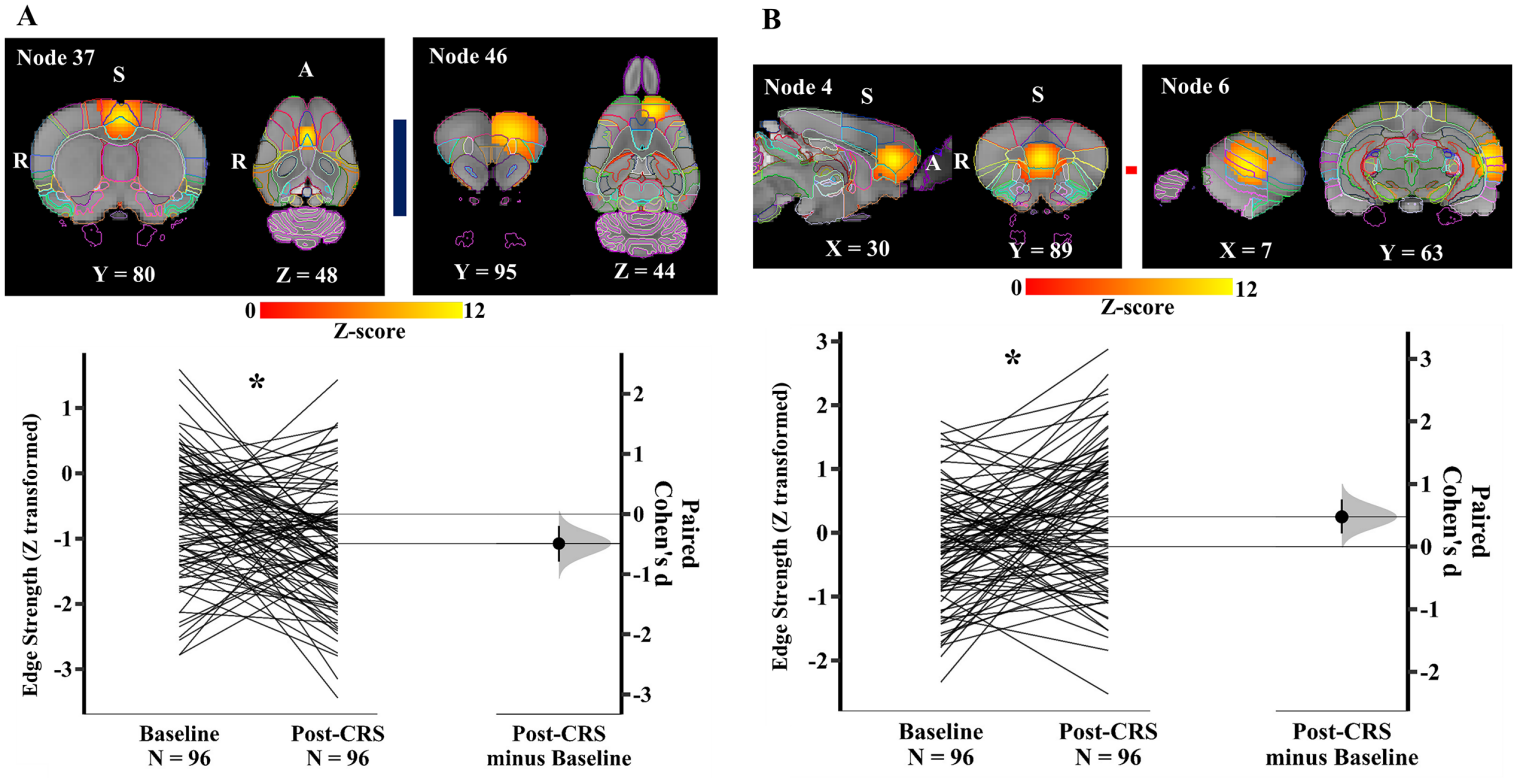
**Significant edge difference following CRS within network 1 (DMN-like network)**. **(A)** Images of the pair of nodes are displayed on the left. Node 37 mainly consisted of bilateral cingulate cortex (posterior part) and node 46 mainly comprised frontal association, orbital, and secondary motor cortex in the left hemisphere. On the right, Gardner-Altman estimation plot showing a significant decrease with medium effect size in the edge strength between node 37 and 46 at post-CRS (* denotes *p* < 0.05). The group-averaged strength was −0.62 and − 1.08 at baseline and post-CRS, respectively. However, there was a high inter-subject variability in the strength changes following CRS. **(B)** Images of another pair of nodes are displayed on the left. Node 4 was a bilateral functional complex comprising infralimbic, prelimbic, and orbital cortex. Node 6 was a unilateral functional complex in the left hemisphere, mainly including auditory, temporal association, and perirhinal cortex. On the right, Gardner-Altman estimation plot showing a significant decrease with medium effect size in the edge strength between node 4 and 6 at post-CRS (* denotes *p* < 0.05). The group-averaged strength was −0.22 and 0.25 at baseline and post-CRS, respectively. However, there was a high inter-subject variability in the strength changes following CRS. The coloured bar joining each pair of nodes indicates the overall group-average connection strength. Longer suggests a stronger connection, red indicates positive, and blue means negative or anti-correlated. A: Anterior; R: Right; S: Superior.

Within the DMN-like network (network 1), a pair of nodes (Figure 6A) showed a significant decrease in the edge/direct connection strength with medium effect size following CRS (baseline: −0.62 ± 0.91; post-CRS: −1.08 ± 0.93; *p* value: 0.040; Cohen’s d: −0.49; 95% CI: −0.79 ~ −0.20). Node 37 mainly consisted of bilateral cingulate cortex (posterior part) and node 46 mainly comprised frontal association, orbital, and secondary motor cortex in the left hemisphere. Moreover, the edge strength between node 4 and 6 (Figure 6B) increased significantly with medium effect size following CRS (baseline: −0.22 ± 0.88; post-CRS: 0.25 ± 1.07; *p* value: 0.029; Cohen’s d: 0.48; 95% CI: 0.21 ~ 0.76). Node 4 was a bilateral functional complex comprising infralimbic, prelimbic, and orbital cortex. Node 6 was a unilateral functional complex in the left hemisphere, mainly including auditory, temporal association, and perirhinal cortex.

**Figure 7:**
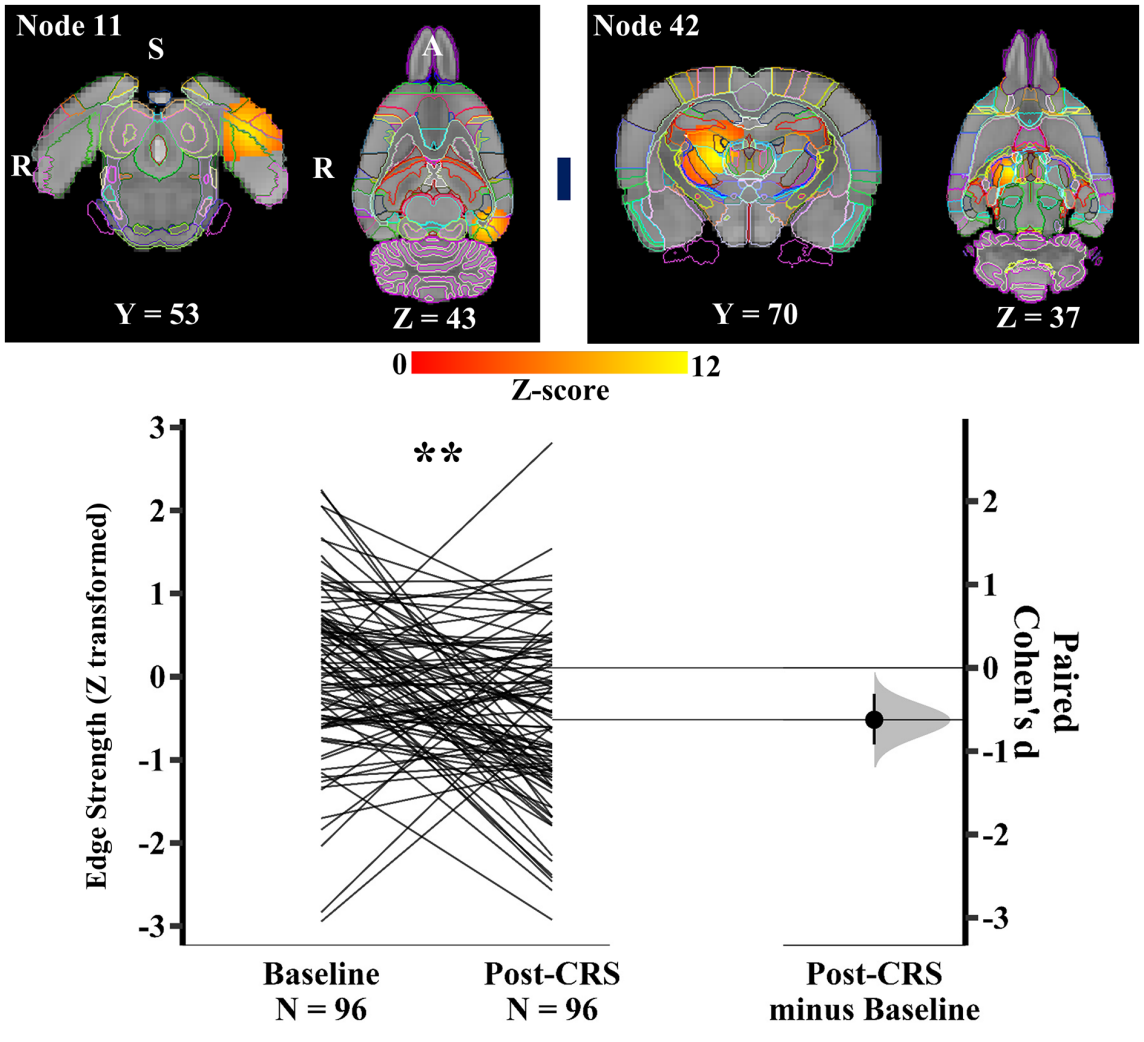
**Significant edge difference following CRS within network 2 (spatial attention-limbic network)**. Images of a pair of nodes are displayed on the left. Node 11 was a functional complex in the left hemisphere, mainly including postrhinal, visual, auditory, temporal association, and medial entorhinal cortex. Node 42 consisted of thalamus with adjacent dentate gyrus and CA3 in the right hemisphere. On the right, Gardner-Altman estimation plot showing a significant decrease with medium effect size in the edge strength between node 11 and 42 at post-CRS (** denotes *p* < 0.01). The group-averaged strength was 0.11 and − 0.52 at baseline and post-CRS, respectively. However, there was a high inter-subject variability in the strength changes following CRS. The coloured bar joining each pair of networks indicates the overall group-average connection strength. Longer suggests a stronger connection, red indicates positive, and blue means negative or anti-correlated. A: Anterior; R: Right; S: Superior.

Within the spatial attention-limbic network (network 2), another pair of nodes (Figure 7) demonstrated a significant decrease in edge strength with medium effect size following CRS (baseline: 0.11 ± 0.99; post-CRS: −0.52 ± 1.02; *p* value: 0.0028; Cohen’s d: -0.62; 95% CI: −0.92 ~ −0.31). Node 11 was a functional complex in the left hemisphere, mainly including postrhinal, visual, auditory, temporal association, and medial entorhinal cortex. Node 42 consisted of thalamus with adjacent dentate gyrus and CA3 in the right hemisphere.

**Figure 8:**
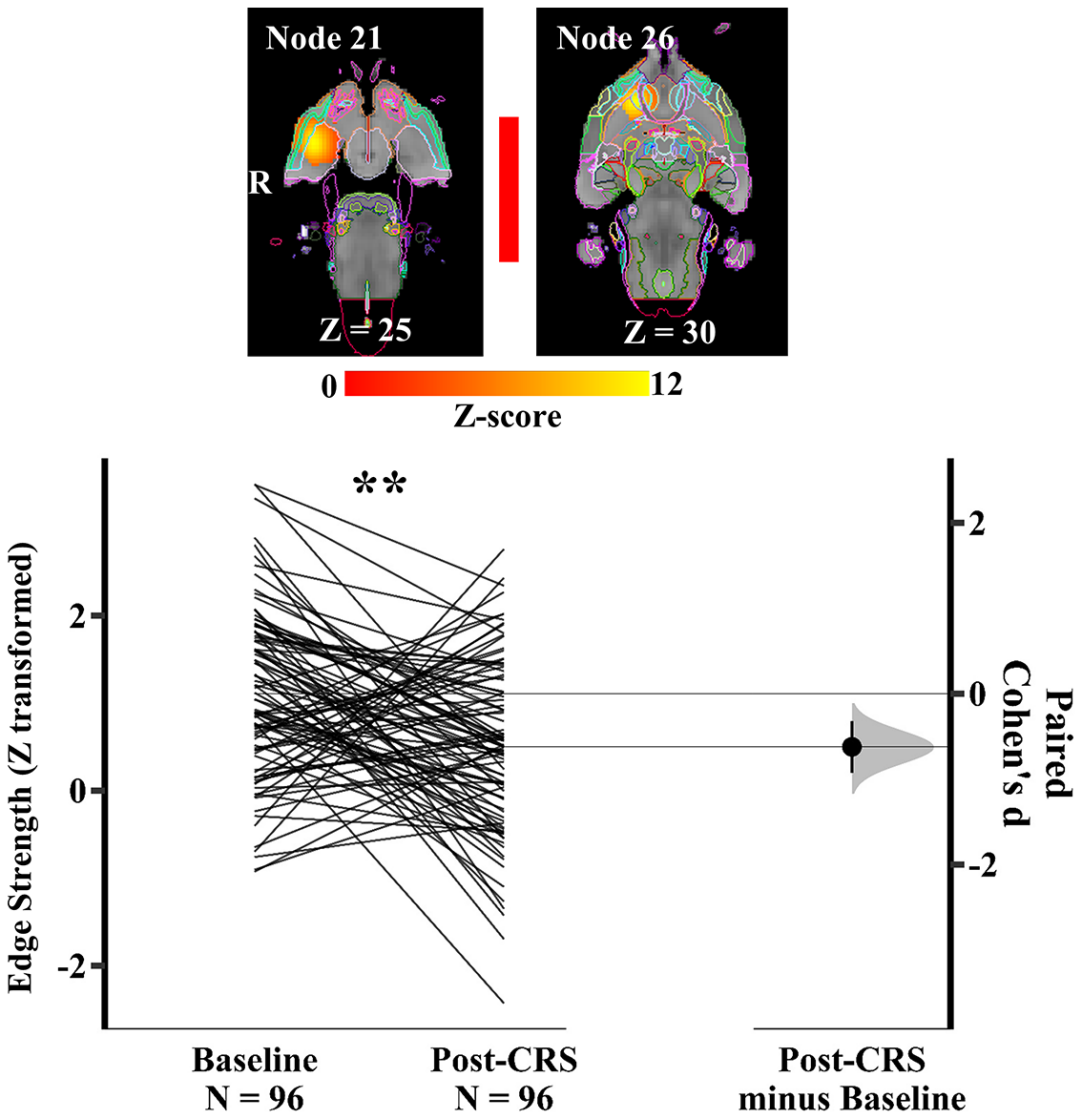
**Significant edge difference following CRS within network 3 (corpus striatum network)**. Images of a pair of nodes are displayed on the left. Node 21 mainly consisted of right amygdala with adjacent piriform cortex. Node 26 mostly comprised nucleus accumbens and ventral pallidum, with their adjacent piriform cortex in the right hemisphere. On the right, Gardner-Altman estimation plot showing a significant decrease with medium effect size in the edge strength between node 21 and 26 at post-CRS (** denotes *p* < 0.01). The coloured bar joining each pair of networks indicates the overall group-average connection strength. Longer suggests a stronger connection, red indicates positive, and blue means negative or anti-correlated. A: Anterior.

Similarly, within the corpus striatum network (network 3), another pair of nodes (Figure 8) presented a significant decrease in the direct connection strength with medium effect size following CRS (baseline: 1.11 ± 0.97; post-CRS: 0.50 ± 0.99; *p* value: 0.0028; Cohen’s d: −0.62; 95% CI: −0.93 ~ −0.32). Node 21 mainly consisted of right amygdala with adjacent piriform cortex. Node 26 mostly comprised nucleus accumbens and ventral pallidum, with their adjacent piriform cortex in the right hemisphere. In other words, the correlation between amygdala and a functional complex (nucleus accumbens and ventral pallidum) in the right hemisphere decreased significantly following CRS.

For the control group (*N* = 13), no changes were detected between baseline and the second scan session (equivalent period to the post-CRS timepoint in the CRS group), except that a pair of nodes (node 5 and 26) presented a significant increase in the direct connection strength at the second scan session (baseline: −0.93 ± 0.78; second scan: 0.15 ± 0.61; *p* value: 0.0444) with strong effect size (Hedges’ g: 1.5; 95% CI: 0.575 ~ 2.14; data not shown). Node 5 mainly consisted of right insular cortex with adjacent perirhinal and secondary somatosensory cortex.

### 3.6. High individual variability in the edge strength within networks before and after CRS

There was a high inter-subject variability in the changes of edge strength within the DMN-like network (network 1) and spatial attention-limbic network (network 2). As shown in Supplementary Material 6, for node 4 and 6, the distribution of changes in the edge strength spanned across all the 11 categories, with 26% (25/96) of the animals demonstrating a change from negative to positive correlation, followed by 11% (11/96) animals changing from negative to less negative correlation. A similar lack of clear direction for the changes in the edge strength was also observed in the pair of nodes 37 and 46, as well as nodes 11 and 42. In contrast, for nodes 21 and 26 within the corpus striatum network (network 3), the majority of changes following CRS demonstrated a decrease from positive correlation, in 64% (61/96) of animals. Additionally, a diversity of edge strength was identified in the pair of nodes 4 and 6 at baseline, with 50% (48/96) of animals presenting negative partial correlation and 32% (31/96) showing positive. A similar diversity was also observed in this pair of nodes at post-CRS, as well as other pairs of abovementioned nodes within the DMN-like and spatial attention-limbic network at both time points.

## 4. Discussion

The main aims of the present study were to use a large dataset to first identify reproducible RSNs in healthy Sprague Dawley rats and then evaluate CRS effects on the functional connectivity in these RSNs within the same animals. The key findings revealed four large-scale networks in the anesthetised rats, including a DMN-like network, a spatial attention-limbic network, a corpus striatum network, and an autonomic network. At a group level, CRS decreased the anticorrelation between the DMN-like network and the autonomic network. In addition, CRS decreased the correlation between the amygdala and a functional complex (nucleus accumbens and ventral pallidum) in the right hemisphere within the corpus striatum network. However, high individual variability in the functional connectivity before and after CRS within RSNs were observed.

### 4.1. Spatial smoothing and RAICAR

The overall increase in the correlation of group-ICA spatial maps along with the increase in the spatial smoothing observed in the present study, is supported by the literature (Fornito et al., 2013; Triana et al., 2020) demonstrating that increasing the magnitude of smoothness can reduce the dissimilarity between voxel time courses. The primary goal of spatial smoothing is to supress spatial noise and improve signal-to-noise ratio. Recent investigations also show that spatial smoothing enhances the functional connection strength at both individual and group level, with a stronger influence at the individual level (Alakorkko et al., 2017; Chen and Calhoun, 2018). Interestingly, the similarly strong correlation (0.77 > *r* > 0.70) at a dimensionality of 10 under four Gaussian kernels may indicate that the cross-correlation analysis by mICA toolbox (Moher Alsady et al., 2016) cannot differentiate the influence of spatial smoothing at a low ICA decomposition dimensionality. This can be explained by the possibility that ICA components at a lower dimensionality (e.g., 10) may be more similar between matched pairs among multiple runs of group-ICA than that components at a higher dimensionality (e.g., over 20) regardless of spatial smoothing.

The present study employs the mICA toolbox (Moher Alsady et al., 2016) to estimate an optimal ICA dimensionality as group-ICA approaches are generally unable to determine the number of components, which is usually achieved empirically. Performing group-ICA with a suboptimal dimensionality can lead to under-fitting or over-fitting of the rs-fMRI data and in turn significantly influence the interpretation of results. Therefore, determining an objective and optimal dimensionality before performing ICA is beneficial. The optimal group-ICA decomposition dimensionality (50 components under Gaussian kernels of FWHM 15.625 mm) chosen for further analysis is simply based on the global maximum recommended by Moher Alsady et al. (2016). However, it would be beneficial to conduct a systematic comparison to evaluate the differences between Gaussian kernels of FWHM 12.5 and 15.625 mm as there were strong correlations (> 0.70) of group-ICA spatial maps across different levels of dimensionality over multiple runs of ICA.

The results from the RAICAR analysis are supported by Pendse et al. (2011) stating that reliable ICA components will be present in almost all ICA runs and in turn will generate tight clusters well distinguishable from the rest. Applying multiple group-ICA runs to groups of subjects with enough diversity is expected to account for the run-to-run variability in ICA approaches both because of the non-convex objective function and inter-subject variability. However, more studies are warranted to ensure reproducible results as only one human study (Pendse et al., 2011) and another rodent study in awake rats (Becerra et al., 2011) have investigated the reproducible RSNs using multiple ICA runs on groups of randomly sampled subjects from a dataset.

### 4.2. Networks

The four large-scale networks classified in the present study show good similarity to the networks observed in healthy humans and rodents in the literature (Becerra et al., 2011; Smith et al., 2013; Zerbi et al., 2015; Bajic et al., 2017; Coletta et al., 2020; Xu et al., 2022).

#### 4.2.1. Network 1 – DMN-like network

The core DMN structures (cingulate, retrosplenial, prelimbic, orbital, and infralimbic cortex) identified here based on the hierarchical analysis are consistent with previously published rodent studies using seed-based approaches or group ICA analysis with a low dimensionality from 10 to 30 (Lu et al., 2012; Stafford et al., 2014; Liska et al., 2015; Sierakowiak et al., 2015; Zerbi et al., 2015; Huang et al., 2016; Grandjean et al., 2020; Whitesell et al., 2021). Hippocampal region (CA) has been suggested as a part of the DMN in some studies (Lu et al., 2012; Zerbi et al., 2015; Huang et al., 2016), but not others (Stafford et al., 2014; Sierakowiak et al., 2015; Grandjean et al., 2020). Although somatosensory structures were shown relatively independent from the DMN in a rodent cohort based on a hierarchical analysis in Zerbi et al. (2015), but not in the present and other rodent studies (Liska et al., 2015; Huang et al., 2016; Whitesell et al., 2021). These inconsistencies may reflect the methodological differences in detecting the DMN. DMN is implicated in cognitive functions, including rumination, self-referential processing at rest, and retrieval of episodic memory in humans (Williams, 2017). DMN has shown the highest activation when individuals are at rest, and it is deactivated when performing goal-directed tasks.

#### 4.2.2. Network 2 – spatial attention-limbic network

The components of this network are similar to what has been observed in awake rodents from other studies (Zhang et al., 2010; Becerra et al., 2011). The colliculi have been well-known to be involved in spatial attention and retrosplenial cortex is considered playing a key function in several cognitive functions, including spatial memory. The asymmetrical resting-state activity of the visual, auditory, and medial entorhinal cortex is consistent with the right-hemispheric lateralization for spatial attention in humans (Agcaoglu et al., 2015; O’Regan and Serrien, 2018).

#### 4.2.3. Network 3 – corpus striatum network

The network is one of the basal ganglia networks observed in previous rodent and human studies (Becerra et al., 2011; Smith et al., 2013; Zerbi et al., 2015). The striatum, insula, orbital cortex, and amygdala forming the cortico–amygdala–striatal circuit mediate emotion processing through collectively processing information in terms of salient stimuli and attentional states in both rodents and primates (Cho et al., 2013; Heilbronner et al., 2016). The asymmetrical resting-state activity of amygdala, CA, and lateral entorhinal cortex in the network agrees with the literature demonstrating a rightwards lateralization of affective processing in humans and primates (Decety and Moriguchi, 2007).

#### 4.2.4. Network 4 – autonomic network

The networks are commonly observed in awake and anesthetized rodents as an ICA component (Hutchison et al., 2010; Liang et al., 2011). The key component of autonomic network – the hypothalamus plays an important role in numerous homeostatic behaviors. The involvement of the hippocampus in the network is considered to be associated with defensive/stress behaviors (Becerra et al., 2011).

### 4.3. Functional connectivity alterations following CRS

The decreased anticorrelation between the whole DMN-like network and subcortical autonomic network in the present study has not previously been reported. Although the mechanism underlying the anticorrelation between the DMN-like and autonomic network is unknown, a meta-analysis study has suggested that these two networks are both associated with autonomic regulation in humans (Beissner et al., 2013). Moreover, studies have reported that the activation of some core structures of the DMN has a downstream inhibitory effect on the activity of hypothalamus and amygdala regarding stress response and negative emotions of anxiety (Etkin et al., 2011; Herman et al., 2016). Disinhibition in the top-down cortical–subcortical circuits may result in excessive stress responses and over-processing of negative emotions, which are associated with anxiety-related disorders (Fridman et al., 2021). The decreased anticorrelation following CRS in the present study can be explained by the top-down disinhibition theory, meaning chronic stress may induce disinhibition from DMN to subcortical autonomic network that regulates stress and anxiety.

This is also the first time that a decreased correlation between the amygdala and a functional complex (nucleus accumbens and ventral pallidum) in the right hemisphere within the corpus striatum network is reported in an animal model. These structures are parts of the reward system, the nucleus accumbens receives glutamatergic inputs from the basolateral amygdala and project GABAergic signals to the ventral pallidum (Hirter et al., 2021). The nucleus accumbens is considered to play a role in one of the core symptoms of depression – anhedonia (Heshmati and Russo, 2015; Liu et al., 2021). For example, the nucleus accumbens has a lower resting-state functional connectivity with some cortical regions in patients with depression. However, the neuroimaging study (Liu et al., 2021) did not analyse the connectivity between amygdala, nucleus accumbens and ventral pallidum. Future studies in investigating the correspondence of functional connectivity between these three subcortical structures in rodent models and patients with depression are necessary.

In contrast with the present results, some human neuroimaging studies have reported that depression is commonly associated with hyperconnectivity within the DMN, as well as aberrant cross-network interaction among DMN, central executive and salience networks (Hamilton et al., 2013; Brakowski et al., 2017). A simple interpretation of the divergent functional connectivity between rodents and humans is that the rodent response to CRS does not reflect the complexity of depression as it is experienced by humans. The limitations of animal models of depression and anxiety-like behavior are beyond the scope of the present article and have been extensively discussed elsewhere (Becker et al., 2021). However, even in humans, findings regarding the direction of functional connectivity alteration within and between human brain networks are still inconclusive because depression is highly heterogenous with various neurobiological substrates (Drysdale et al., 2017). The present study also reveals that there is high inter-subject variability of functional connectivity at baseline and post-CRS in rats as well, suggesting that it may be possible to classify rodent neural phenotypes to refine the connectivity analysis. Future studies should investigate rodent phenotypes using not only brain connectivity but also behavioural measures, potentially providing opportunity to model tailored approaches to prevention and treatment of neuropsychiatric conditions including depression.

### 4.4. Limitations and future direction

Additional limitations are worth mentioning. First, only male late adolescent/young adult Sprague–Dawley rats were used in the analysis due to data availability. This means that the brains of these animals were still developing during the CRS intervention and may have confounded interpretation of the network changes (Hennessy et al., 2022). However, this age range is relevant to studying mood disorders in young people. It is expected that the present approach of using large datasets will lead to a better understanding of how the developing brain is impacted by environmental stressors, and how this may lead to long-term changes in mood regulation. Future studies including female rats, aged rats, other inbred and outbred strains will be necessary to assess the generalizability of present results.

Moreover, the level of anaesthesia is a key consideration in MRI studies and the present study applied combined use of isoflurane and medetomidine at doses, which have been found to provide stable anaesthesia with the least impact on brain activity (Grandjean et al., 2014; Bukhari et al., 2017; Paasonen et al., 2018; Grandjean et al., 2023). At the time the experiments in this study were carried out, a standard time for the change of anaesthesia before rs-fMRI scanning was not established. Rather, individual animals’ breathing rate was employed as an indicator because individual animals have been found to vary in their response to isoflurane and medetomidine (Seewoo et al., 2020). Recently, a standard 45-min period in mice has been recommended after switching from isoflurane alone to the combined regimen of isoflurane and medetomidine (ISO/MED; Pradier et al., 2021). However, considering the experimental design, it is not clear whether differences in brain activity between isoflurane alone (at 1%) and three combined regimes (ISO-0.6%/MED-25 min, ISO-0.2%/MED-45 min, ISO-0.2%/MED-100 min) resulted from the reduction in the isoflurane concentration or waiting period following the induction of medetomidine. While additional study on time-dependency of anaesthetic on brain activity is needed, the consistent timing (32 min ± 4) of the combined anaesthetic protocol employed in the present study allows confident interpretation of results.

In terms of analysis, the parcellation approach to define the spatial boundary of functional nodes may be too simple and can be improved with more sophisticated approaches (e.g., advanced clustering methods) to address the uncertainty resulting from regions where multiple nodes overlap and *Z*-scores are low. Finally, only hierarchical clustering analysis was applied to classify the networks. Future efforts in systematically comparing the differences on the RSN classification with other algorithms, such as the fuzzy-c-means clustering algorithm (Lee et al., 2012) and deep learning algorithms like Siamese ICA (Chou et al., 2022), are necessary.

## 5. Conclusion

The present study is the first to construct reproducible RSNs in anaesthetized rats through identifying optimal and reproducible functional components, and in turn evaluate functional connectivity changes in the RSNs following a CRS model within the same animals. The key findings revealed four large-scale networks that are homologous across species, decreased anticorrelation between DMN-like and autonomic network, decreased correlation between amygdala and a functional complex (nucleus accumbens and ventral pallidum) in the right hemisphere within the corpus striatum network. Moreover, high inter-subject variability of functional connectivity is observed within networks, indicating rats may demonstrate different neural phenotypes as humans. Therefore, future efforts in classifying neural phenotypes in rodents might improve the sensitivity and translational impact of models used to address aetiology and treatment of psychiatric conditions including depression.

## Data availability statement

The original contributions presented in the study are included in the article/Supplementary material, further inquiries can be directed to the corresponding author.

## Ethics statement

The animal study was reviewed and approved by University of Western Australia Animal Ethics Committee (RA/3/100/1640).

## Author contributions

TD and JR conceived and designed the study. LH and SB collected a subset of data that is not previously published. TD carried out the analysis with guidance from BS and TR, and wrote the first draft of the manuscript. All authors contributed to the article and approved the submitted version.

## Funding

JR is supported by a Senior Fellowship from MSWA and the Perron Institute for Neurological and Translational Science. TD is supported by the Australian Government International Research Training Program scholarship, and Byron Kakulas Prestige scholarship.

## Conflict of interest

The authors declare that the research was conducted in the absence of any commercial or financial relationships that could be construed as a potential conflict of interest.

## Publisher’s note

All claims expressed in this article are solely those of the authors and do not necessarily represent those of their affiliated organizations, or those of the publisher, the editors and the reviewers. Any product that may be evaluated in this article, or claim that may be made by its manufacturer, is not guaranteed or endorsed by the publisher.
