# Supplementary material for: Identifying reproducible resting state networks and functional connectivity alterations following chronic restraint stress in anaesthetized rats
Source: Front Neurosci. 2023 May 22;17:1151525. doi: 10.3389/fnins.2023.1151525 (PMC10239969; doi:10.3389/fnins.2023.1151525)
Supplement: Supplementary file 1 [file Data_Sheet_1.ZIP › Supplementary_Material_1_Workflow_Scripts.pdf]

Supplementary Script

1. Common pre-processing steps

1.1. Bruker data (PvDatasets) Extraction

```
#!/bin/sh
for folder in /PATH/TO/Baseline_VS_CRS/*_MRI_Raw/*/; do [ -d "$folder" ] && cd "$folder" &&
 unzip '*.PvDatasets'
done
```

1.2. Convert DICOM into NifTi

```
#!/bin/sh
module load dcm2niix
for folder in /PATH/TO/Baseline_VS_CRS/*_MRI_Raw/*/; do
		[ -d "$folder" ] && cd "$folder" &&
dcm2niix -f %n_%p_%s_%t -z y -o ./ "$folder"
done
```

1.3. Reorient images in the radiological view

```
#!/bin/sh
module load fsl/6.0.3
for folder in /PATH/TO/Baseline_VS_CRS/Cohort*/*/C*/*/t2_coronal/; do
   [ -d "$folder" ] && cd "$folder" &&
#reorient anatomical images
cp *TurboRARE*.nii.gz t2.nii.gz
fslorient -setsform -0.1 0 0 0 0 0 1.05 0 0 -0.1 0 0 0 0 0 1 t2.nii.gz
fslorient -copysform2qform t2.nii.gz
fslswapdim t2.nii.gz x z -y t2.nii.gz
#reorient functional images
cp *EPI*.nii.gz rs.nii.gz
fslorient -setsform -0.3 0 0 0 0 0 1.05 0 0 -0.3 0 0 0 0 0 1 rs.nii.gz
fslorient -copysform2qform rs.nii.gz
fslswapdim rs.nii.gz x z -y rs.nii.gz
done
```

1.4. Correct bias field signals

```
#!/bin/sh
for folder in /PATH/TO/Baseline_VS_CRS/Cohort*/*/C*/*/ t2_coronal/; do
		[ -d "$folder" ] && cd "$folder" &&
/usr/local/3dslicer/4.8.1/Slicer --launch /usr/local/3dslicer/4.8.1/lib/Slicer-4.8/cli-
modules/N4ITKBiasFieldCorrection --meshresolution 1,1,1 --splinedistance 0 --bffwhm 0 --
iterations 50,40,30 --convergencethreshold 0.0001 --bsplineorder 3 --shrinkfactor 4 --
wienerfilternoise 0 --nhistogrambins 0 t2.nii.gz t2_BFC.nii.gz
done
```

1.5. Skull striping for anatomical images and create an individualised brain mask

```
#!/bin/sh
module load fsl/6.0.3
for folder in /PATH/TO/Baseline_VS_CRS/Cohort*/*/C*/*/t2_coronal/; do
        [ -d "$folder" ] && cd "$folder" &&
flirt -in t2_BFC.nii.gz -ref /PATH/TO/Atlas/atlas_brain.nii.gz -out t2_hratlas_brain.nii.gz -omat
t2_hratlas_brain.mat -bins 30 -cost corratio -searchrx -90 90 -searchry -90 90 -searchrz -90 90 -
dof 9  -interp trilinear

#get inverse matrix
convert_xfm -omat t2_hratlas_brain_inverse.mat -inverse t2_hratlas_brain.mat
#get brain mask in t2 space and extract t2
flirt -in /PATH/TO/Atlas/atlas_brain.nii.gz -ref t2_BFC.nii.gz -out t2_hratlas_brain_inverse.nii.gz -
init t2_hratlas_brain_inverse.mat -applyxfm
fslmaths t2_hratlas_brain_inverse.nii.gz -bin t2_hratlas_brain_inverse_mask.nii.gz
fslmaths t2_BFC.nii.gz -mas t2_hratlas_brain_inverse_mask.nii.gz t2_brain.nii.gz
rm t2_hratlas_brain.nii.gz

#upscale voxel sizes
cp t2_brain.nii.gz t2_brain_x10_temp.nii.gz
fslchpixdim t2_brain_x10_temp.nii.gz 1 10.5 1

# co-register to downsampled atlas
flirt -in t2_brain_x10_temp.nii.gz -ref  '/PATH/TO/Atlas/atlas_downsampled8.nii.gz' -out
t2_brain_x10_atlas.nii.gz -omat t2_brain_x10_atlas.mat -bins 30 -cost corratio -searchrx -90 90 -
searchry -90 90 -searchrz -90 90 -dof 9  -interp trilinear

#get reverse matrix and ensure no extra-brain matter remains
convert_xfm -omat t2_brain_x10_atlas_inverse.mat -inverse t2_brain_x10_atlas.mat
flirt -in /PATH/TO/Atlas/atlas_downsampled8.nii.gz -ref t2_brain_x10_temp.nii.gz -out
t2_brain_x10_atlas_inverse.nii.gz -init t2_brain_x10_atlas_inverse.mat -applyxfm
fslmaths t2_brain_x10_atlas_inverse.nii.gz -bin t2_brain_x10_atlas_inverse_mask.nii.gz
fslmaths t2_brain_x10_temp.nii.gz -mas t2_brain_x10_atlas_inverse_mask.nii.gz
t2_brain_x10.nii.gz

cp t2_brain_x10.nii.gz t2_brain_temp.nii.gz
fslchpixdim t2_brain_temp.nii.gz 0.1 1.05 0.1
fslmaths t2_brain_temp.nii.gz -bin t2_mask.nii.gz
done
```

1.6. Brain extraction for functional images

```
#!/bin/sh
module load fsl/6.0.3
for folder in /PATH/TO/Baseline_VS_CRS/Cohort*/*/C*/*/t2_coronal/; do
        [ -d "$folder" ] && cd "$folder" &&
flirt -in rs.nii.gz -ref t2.nii.gz -out rs_t2.nii.gz -omat rs_t2.mat -bins 30 -cost corratio -searchrx 0 0
-searchry 0 0 -searchrz 0 0 -dof 6 -interp trilinear
fslmaths rs_t2.nii.gz -mas t2_mask.nii.gz rs_t2_brain.nii.gz
convert_xfm -omat rs_t2_inverse.mat -inverse rs_t2.mat
flirt -in rs_t2_brain.nii.gz -ref rs.nii.gz -out rs_t2_brain_inverse.nii.gz -init rs_t2_inverse.mat -
applyxfm
fslmaths rs_t2_brain_inverse.nii.gz -bin rs_mask.nii.gz
fslmaths rs.nii.gz -mas rs_mask.nii.gz rs_brain.nii.gz
done
```

1.7. Increase image resolution by a factor of 10

```
#!/bin/sh
module load fsl/6.0.3
for folder in /PATH/TO/Baseline_VS_CRS/Cohort*/*/C*/*/ t2_coronal/; do
        [ -d "$folder" ] && cd "$folder" &&
#upscale voxel sizes
cp rs_brain.nii.gz rs_brain_x10.nii.gz
fslchpixdim rs_brain_x10.nii.gz 3 10.5 3
done
```

1.8. Quality inspection

```
#!/bin/sh
module load fsl/6.0.3
for file in / PATH/TO/Baseline_VS_CRS/Cohort*/*/C*/*/t2*/t2_brain.nii.gz; do
#get animal ID
ID=${file##*Baseline_VS_CRS/}
ID=${ID%/t2_*}
ID=${ID////_}
echo $ID
slices "$file" -o /PATH/TO/Baseline_VS_CRS/t2_brain_inspection/"$ID"
done

for file in / PATH/TO/Baseline_VS_CRS/Cohort*/*/C*/*/t2*/rs_brain.nii.gz; do
#get animal ID
ID=${file##*Baseline_VS_CRS/}
ID=${ID%/rs_*}
ID=${ID////_}
echo $ID
slices "$file" -o /PATH/TO/Baseline_VS_CRS/rs_brain_inspection/"$ID"
done
```

2. Further functional image pre-processing

Single-session ICA was performed on the FSL/MELODIC GUI without a script.

2.1. FIX training

```
#!/bin/sh
module load fsl/6.0.3
module load R
module load matlab
mkdir FIX_training  && cd FIX_training
## Manually label single-session ICA’s decomposition of 50 datasets into signal or noise into txt tile named “hand_labels_noise.txt”
ls /PATH/TO/Baseline_VS_CRS/Cohort*/*/*/*/t2_coronal/rs_brain_x10+.ica/hand_labels_noise.txt > All_labels.txt (##delete /hand_labels_noise.txt in the txt file)
## Train a dataset based on all hand-labelled files.
/usr/local/fix/1.068/bin/fix -t FIX_training.RData `cat All_labels.txt`
```

2.2. Apply FSL/FIX to clean EPI data after single-session ICA && atlas registration

```
#!/bin/sh
module load fsl/6.0.3
for folder in /PATH/TO/Baseline_VS_CRS/Cohort*/*/C*/*/ t2_coronal/; do
        [ -d "$folder" ] && cd "$folder" &&
echo running FIX on "$folder"
/usr/local/fix/1.068/bin/fix rs_brain_x10.ica /PATH/TO/Atlas/training.RData 20

# Atlas registration
flirt -in filtered_func_data_clean.nii.gz -ref /PATH/TO/Atlas/atlas_downsampled8.nii.gz -out filtered_func_data_clean_t2_atlas.nii.gz -applyxfm -init reg/example_func2standard.mat -interp trilinear
done
```

3. Group-level ICA

3.1. Atlas mask generation for the ROI

Extract masks from WHS_SD_rat_atlas based on their atlas label ID using convert3d. The following commands are examples of extracting high resolution masks for CA3 and Dendate gyrus. Then you can combine these masks to generate desired ROI masks using fslmaths.

```
#!/bin/sh
module load convert3d
mkdir /PATH/TO/Atlas/ WHS_SD_rat_atlas_v4_masks
cd /PATH/TO/Atlas/ WHS_SD_rat_atlas_v4_masks
c3d WHS_SD_rat_atlas_v4.nii.gz -thresh 95 95 1 0 -o CA3_mask.nii.gz
c3d WHS_SD_rat_atlas_v4.nii.gz -thresh 96 96 1 0 -o Dentate_gyrus_mask.nii.gz
```

Resample the high-resolution masks to low-resolution masks

```
#!/bin/sh
module load fsl/6.0.3
mkdir /PATH/TO/Atlas/low_resolution_masks && cd /PATH/TO/Atlas/low_resolution_masks
for file in /PATH/TO/Atlas/WHS_SD_rat_atlas_v4_masks/*_mask.nii.gz; do
mask=${file%"_mask.nii.gz"}
mask=${mask#"/PATH/TO/Atlas/WHS_SD_rat_atlas_v4_masks/"}
cp "$file" "$mask"_lrmask.nii.gz
fslchpixdim "$mask"_lrmask.nii.gz 0.39 0.39 0.39
fslorient -setsform 0.39 0 0 -9.31 0 0.39 0 -24.36 0 0 0.39 -8.88 0 0 0 1 "$mask"_lrmask.nii.gz
fslorient -copysform2qform "$mask"_lrmask.nii.gz
flirt -in "$file" -ref  '/ PATH/TO /Atlas/atlas_downsampled8.nii.gz' -out "$mask"_lrmask.nii.gz -
applyxfm -nosearch -usesqform -noresampblur
fslmaths "$mask"_lrmask.nii.gz -bin "$mask"_lrmask.nii.gz
done
## create the ROI mask
fslmaths lrmask.nii.gz -thr 2 ROIs.nii.gz
for mask in ../ROIs_mask/*lrmask.nii.gz; do fslmaths ROIs.nii.gz -add $mask ROIs.nii.gz; done
fslmaths ROIs.nii.gz -bin ROIs.nii.gz
```

Make unilateral masks from above bilateral masks and then resample the bilateral masks to low-resolution masks using the same method mentioned above.

```
#!/bin/sh
module load fsl/6.0.3
for file in /PATH/TO/Atlas/WHS_SD_rat_atlas_v4_masks/*_mask.nii.gz; do
mask=${file%"_mask.nii.gz"}
mask=${mask#"/PATH/TO/Atlas/WHS_SD_rat_atlas_v4_masks/"}
fslroi $file "$mask"_left_mask.nii.gz 0 244 0 1025 0 513
flirt -in "$file" -ref '/PATH/TO/Atlas/WHS_SD_rat_atlas_v4.nii.gz' -out "$mask"_left_mask.nii.gz  -
applyxfm -dof 6 -nosearch -usesqform
fslmaths $file -sub "$mask"_left_mask.nii.gz "$mask"_right_mask.nii.gz
done
```

3.2. Spatial smoothing effect on optimal group-ICA dimensionality

The part was mostly performed on the mICA toolbox GUI. However, the original python codes for generating cross-correlation matrix and hungarian sorting only worked under python 2 environment. Therefore, the python 2 code (ic_corr.py) was modified to work under python 3 environment. Changes to the original codes were presented below.

A). Line 31:

```
import sys, math, os, re, subprocess
```

changed to

```
    from builtins import str
    from builtins import range
    from past.utils import old_div
    import sys, math, os, re, subprocess
```

B). Line 69 – 75:

```
for dim in dimsstr_split:
    if dim.find("-") > -1:
        minmax = dim.split("-")
        dim=range(int(minmax[0]),int(minmax[1])+1)
        dims = dims + dim
    else:
        dims.append(int(dim))
```

changed to

```
for dim in dimsstr_split:
    if dim.find("-") > -1:
        minmax = dim.split("-")
        dim=list(range(int(minmax[0]),int(minmax[1])+1))
        dims = dims + dim
    else:
        dims.append(int(dim))
```

C). Line 102:

```
if int(proc1.stdout.read().rstrip('\n')) != 1 or int(proc2.stdout.read().rstrip('\n')) != 1 or
int(n1.stdout.read().rstrip('\n')) != int(dim) or int(n2.stdout.read().rstrip('\n')) != int(dim):
```

changed to

```
if int(proc1.stdout.read().rstrip('\n'.encode())) != 1 or
int(proc2.stdout.read().rstrip('\n'.encode())) != 1 or int(n1.stdout.read().rstrip('\n'.encode())) !=
int(dim) or int(n2.stdout.read().rstrip('\n'.encode())) != int(dim):
```

D). Line 177-179:

```
if len(dims) < 10:
    xtics=range(0,len(dims))
else:
    xtics=range(0,len(dims), int(math.ceil(len(dims)/15.0)))
```

changed to

```
if len(dims) < 10:
    xtics=list(range(0,len(dims)))
else:
    xtics=list(range(0,len(dims), int(math.ceil(len(dims)/15.0))))
```

3.3. RAICAR

Several changes to gRAICAR codes (gRAICAR_step3.m) were done to match the spatial resolution (3.125 × 3.125 × 3.125 mm^3) of our dataset.

```
module load fsl/6.0.3
module load matlab/r2019b
cd /PATH/TO/Baseline_VS_CRS/Analysis_ICA/RAICAR_reproducibility/mICA_ROIs5
#Make a list for all melodic_IC
ls sample*/group*/dim50/melodic_IC.nii.gz > group_ROIs5_dim50.list

#Create group mask
for i in {02..50}; do fslmaths sample_0001/group1/dim50/mask.nii.gz -mul
sample_00"$i"/group1/dim50/mask.nii.gz mask_group_dim50.nii.gz;done
for i in {01..50}; do fslmaths mask_group_dim50.nii.gz -mul
sample_00"$i"/group2/dim50/mask.nii.gz -bin mask_group_dim50.nii.gz;done

#Running gRAICAR on the terminal
matlab -nodisplay -nosplash -r "run('../gRAICAR_ROIs5_dim50.m');"
#Matlab script of ‘gRAICAR_ROIs5_dim50.m':
addpath (genpath('/ PATH/TO/Baseline_VS_CRS/Analysis_ICA/RAICAR_reproducibility/gRAICAR-
master'));
addpath('/ PATH/TO/Baseline_VS_CRS/Analysis_ICA/RAICAR_reproducibility/mICA_ROIs5/');
settings.workdir = '/
PATH/TO/Baseline_VS_CRS/Analysis_ICA/RAICAR_reproducibility/mICA_ROIs5/';
settings.outdir = '
/PATH/TO/Baseline_VS_CRS/Analysis_ICA/RAICAR_reproducibility/mICA_ROIs5/RAICAR_ROIs5_di
m50';
settings.subjlist = '/
PATH/TO/Baseline_VS_CRS/Analysis_ICA/RAICAR_reproducibility/mICA_ROIs5/group_ROIs5_dim
50.list';
settings.maskpath = '/
PATH/TO/Baseline_VS_CRS/Analysis_ICA/RAICAR_reproducibility/mICA_ROIs5/mask_group_dim5
0.nii.gz';
settings.taskname = 'ROIs5_dim50';
settings.ncores = 1;
settings.useRAICAR  = 0;
settings.icapath  = '/
PATH/TO/Baseline_VS_CRS/Analysis_ICA/RAICAR_reproducibility/mICA_ROIs5/sample_0001/gro
up1/dim50/melodic_IC.nii.gz';
settings.savemovie = 0;
settings.webreport = 1;
settings.displayThreshold = 1.5;
settings.comPerPage = 10;
[pass, settings] = gRAICAR_check_settings (settings);
[status, exeption] = gRAICAR_step1(settings);
[status, exeption] = gRAICAR_step2(settings);
[status, exeption] = gRAICAR_step3(settings);
```

4. Network modelling

```
module load fsl/6.0.3
module load matlab/r2019b
cd /PATH/TO/Baseline_VS_CRS/Analysis_ICA/RAICAR_reproducibility/mICA_ROIs5/
RAICAR_ROIs5_dim50
fslmerge -t melodic_IC.nii.gz compMaps/comp{001..50}.nii
### Nodes parcellation
echo "1" > grot_dummy.txt
melodic -i melodic_IC.nii.gz --ICs=melodic_IC.nii.gz --mix=grot_dummy.txt -o melodic_Zstat --Oall -
-report -v --mmthresh=0.5

# rename thresholded ICs using leading zeros to avoid wrong order
for i in {1..50}; do immv melodic_Zstat/stats/thresh_zstat"${i}".nii.gz
melodic_Zstat/stats/thresh_zstat`printf "%04g\n" $i`.nii.gz; done
fslmerge -t all_thresh_zstat.nii.gz melodic_Zstat/stats/thresh_zstat*.nii.gz
fslmaths melodic_IC.nii.gz -Tstd -bin mask.nii.gz
fslmaths mask.nii.gz -bin -thr 2 empty.nii.gz
fslmerge -t all_thresh_zstat_plusempty.nii.gz empty.nii.gz all_thresh_zstat.nii.gz
fslmaths all_thresh_zstat_plusempty.nii.gz -Tmaxn parcellation.nii.gz

#check if any parcellated node is empty
for i in {1..50}; do c3d parcellation.nii.gz -thresh "$i" "$i" 1 0 -o melodic_Zstat/stats/mask_`printf
"%04g\n" $i`.nii.gz;done
for i in {1..50};do echo "$i" && fslmaths compMaps/comp`printf "%03g\n" $i`.nii.gz -mas
melodic_Zstat/stats/mask_`printf "%04g\n" $i`.nii.gz melodic_Zstat/stats/node`printf "%04g\n"
$i`.nii.gz;done

fslmerge -t Parcellation_IC.nii.gz melodic_Zstat/stats/node{0001..0050}.nii.gz
slices_summary Parcellation_IC 3 / PATH/TO/Baseline_VS_CRS/Analysis_ICA/bg_image.nii.gz
Parcellation_IC.sum -1
#Apply dual regression to extract subject-specific time series for all baseline data
dual_regression Parcellation_IC.nii.gz 1 -1 0 ROIs5_baseline_dim50.dr `cat ../input_preproc.txt`

#The following scripts were performed on the matlab GUI
addpath
/PATH/TO/Baseline_VS_CRS/Analysis_ICA/RAICAR_reproducibility/mICA_ROIs5/RAICAR_ROIs5_di
m50;
addpath /PATH/TO/FSLnetwork_analysis/FSLNets;
addpath(sprintf('%s/usr/local/fsl/6.0.3/etc/matlab',getenv('fsl')));
addpath /PATH/TO/FSLnetwork_analysis/L1precision;
group_maps = ' Parcellation_IC';
baseline_ts_dir = 'ROIs5_baseline_dim50.dr';
baseline_ts = nets_load(baseline_ts_dir, 1.5, 0);
baseline_ts_spectra = nets_spectra(baseline_ts);
baseline_ts.DD = [1:50];
```

Continued

```
baseline_Full = nets_netmats(baseline_ts,1,'corr');
[baseline_Znet_F,baseline_Mnet_F]=nets_groupmean(baseline_Full,0);
nets_hierarchy(baseline_Znet_F,baseline_Znet_P, baseline_ts.DD,group_maps);

#Merge nodes into 4 networks based on the hierarchical clustering
for i in {1..4}; do cp Networks_empty.nii.gz Network"$i"_add.nii.gz;done
#Network1 14ICs
for i in {0037,0050,0009,0023,0010,0016,0035,0044,0047,0006,0043,0004,0046,0034}; do echo
"$i" && fslmaths Network1_add.nii.gz -add melodic_Zstat/stats/node"$i"
Network1_add.nii.gz;done
fslmerge -t Network1.nii.gz
melodic_Zstat/stats/node{0037,0050,0009,0023,0010,0016,0035,0044,0047,0006,0043,0004,004
6,0034}.nii.gz

#Network2 14ICs
for i in {0007,0030,0036,0048,0011,0014,0022,0040,0015,0039,0041,0027,0031,0042}; do echo
"$i" && fslmaths Network2_add.nii.gz -add melodic_Zstat/stats/node"$i"
Network2_add.nii.gz;done
fslmerge -t Network2.nii.gz
melodic_Zstat/stats/node{0007,0030,0036,0048,0011,0014,0022,0040,0015,0039,0041,0027,003
1,0042}.nii.gz

#Network3 15ICs
for i in {0001,0024,0025,0029,0002,0026,0033,0028,0005,0021,0032,0017,0020,0038,0049}; do
echo "$i" && fslmaths Network3_add.nii.gz -add melodic_Zstat/stats/node"$i"
Network3_add.nii.gz;done
fslmerge -t Network3.nii.gz
melodic_Zstat/stats/node{0001,0024,0025,0029,0002,0026,0033,0028,0005,0021,0032,0017,002
0,0038,0049}.nii.gz

#Network4 7ICs
for i in {0003,0012,0008,0013,0018,0045,0019}; do echo "$i" && fslmaths Network4_add.nii.gz -
add melodic_Zstat/stats/node"$i" Network4_add.nii.gz;done
fslmerge -t Network4.nii.gz
melodic_Zstat/stats/node{0003,0012,0008,0013,0018,0045,0019}.nii.gz

fslmerge -t Networks_4clusters.nii.gz Network{1..4}_add.nii.gz

## Prepare slices summary for later use
for file in Network*.nii.gz; do echo $file && name=${file%".nii.gz"} && slices_summary $file 3
/PATH/TO/Baseline_VS_CRS/Analysis_ICA/atlasX10_downsampled8.nii.gz "$name".sum -1;done
```

Continued

```
###Volume and Avg_Z scores extraction for each network
for i in {1..4}; do echo "$i" && for mask in
/PATH/TO/Baseline_VS_CRS/Analysis_ICA/ROIs_mask/*_lrmask.nii.gz; do echo "$mask" &&
stats=$(fslstats Network"$i"_add.nii.gz -k "$mask" -M -V) && filename=${mask%"_lrmask.nii.gz"}
&& filename=${filename#"/PATH/TO/Baseline_VS_CRS/Analysis_ICA/ROIs_mask/"} && echo -e
Network"$i" $filename $stats >> Networks_avgZ_volume.xls;done;done

###Volume and Avg_Z scores extraction for each node within each network
mkdir Network{1..4}
for i in {0037,0050,0009,0023,0010,0016,0035,0044,0047,0006,0043,0004,0046,0034}; do echo
"$i" && for file in melodic_Zstat/stats/node"$i".nii.gz;do cp $file Network1;done;done

for i in {0007,0030,0036,0048,0011,0014,0022,0040,0015,0039,0041,0027,0031,0042}; do echo
"$i" && for file in melodic_Zstat/stats/node"$i".nii.gz;do cp $file Network2;done;done

for i in {0001,0024,0025,0029,0002,0026,0033,0028,0005,0021,0032,0017,0020,0038,0049}; do
echo "$i" && for file in melodic_Zstat/stats/node"$i".nii.gz;do cp $file Network3;done;done

for i in {0003,0012,0008,0013,0018,0045,0019}; do echo "$i" && for file in
melodic_Zstat/stats/node"$i".nii.gz;do cp $file Network4;done;done

for i in {1..4}; do echo Network"$i" && for node in Network"$i"/node*.nii.gz; do echo $node &&
nodename=${node%".nii.gz"} && for mask in
/PATH/TO/Baseline_VS_CRS/Analysis_ICA/ROIs_mask/*_lrmask.nii.gz; do echo "$mask" &&
region=${mask%"_lrmask.nii.gz"} &&
region=${region#"/PATH/TO/Baseline_VS_CRS/Analysis_ICA/ROIs_mask/"} && stats=$(fslstats
$node -k $mask -M -V) && echo -e Network"$i" $nodename $region $stats >>
Nodes50_avgZ_volume.xls;done;done;done
```

5. Comparison of CRS and control groups to baseline

5.1. Identify differences between networks

```
module load fsl/6.0.3
module load matlab/r2019b
cd /PATH/TO/Baseline_VS_CRS/Analysis_ICA/RAICAR_reproducibility/mICA_ROIs5_BvD/dim50
#Apply dual regression to extract subject-specific time series for the CRS group at two timepoints
dual_regression Networks_4clusters.nii.gz 1 -1 0 mICA_ROIs5_BvD.dr `cat ../input_preproc.txt`

#Matlab GUI with FSLNet
addpath /PATH/TO/Baseline_VS_CRS/Analysis_ICA/RAICAR_reproducibility/mICA_ROIs5/
mICA_ROIs5_BvD/dim50;
addpath /PATH/TO/FSLnetwork_analysis/FSLNets;
addpath(sprintf('%s/usr/local/fsl/6.0.3/etc/matlab',getenv('fsl')));
addpath /PATH/TO/FSLnetwork_analysis/L1precision;
ts_dir = 'ROIs5_BvD_dim50.dr';
ts = nets_load(ts_dir, 1.5, 0);
group_maps = 'Networks_4clusters';
ts.DD = [1:4];
ts = nets_tsclean(ts,0);
All_Pridgep_nets = nets_netmats(ts,1,'ridgep',0.1);
[All_Znet_P,All_Mnet_P]=nets_groupmean(All_Pridgep_nets,1);
[p_uncorrected,p_corrected]=nets_glm(All_Pridgep_nets,'design.mat','design.con',1);
nets_edgepics(ts,group_maps, All_Znet_P,reshape(p_corrected(1,:),ts.Nnodes,ts.Nnodes),2);
nets_edgepics(ts,group_maps, All_Znet_P,reshape(p_corrected(2,:),ts.Nnodes,ts.Nnodes),2);
nets_boxplots(ts, All_Pridgep_nets,1,3,-1);
nets_boxplots(ts, All_Pridgep_nets,1,4,-1);

#extract edge strength value on matlab
IC1=3;
IC2=1;
i=(IC1-1)*ts.Nnodes + IC2;
Ngroup1=ts.Nsubjects/2;
Baseline_edge= All_Pridgep_nets(1:Ngroup1,i);
Post-CRS_edge = All_Pridgep_nets(Ngroup1+1:end,i);

## Same above steps repeated for the control group after
cd /PATH/TO/Baseline_VS_CRS/Analysis_ICA/RAICAR_reproducibility/mICA_ROIs5_Control/dim50
```

5.2. Identify differences within each network

```
module load fsl/6.0.3
module load matlab/r2019b
cd /PATH/TO/Baseline_VS_CRS/Analysis_ICA/RAICAR_reproducibility/mICA_ROIs5_BvD/dim50
#Apply dual regression to extract subject-specific time series for the CRS group at two timepoints
for i in {1..8}; do echo "$i" && slices_summary Network"$i" 3
/PATH/TO/Baseline_VS_CRS/Analysis_ICA/bg_image_SBA.nii.gz Network"$i".sum -1;done
for i in {1..8}; do echo "$i" && dual_regression Network"$i".nii.gz 1 -1 0 Network"$i".dr `cat
../input_preproc.txt`

#Matlab GUI with FSLNet
addpath /PATH/TO/Baseline_VS_CRS/Analysis_ICA/RAICAR_reproducibility/mICA_ROIs5/
mICA_ROIs5_BvD/dim50;
addpath /PATH/TO/FSLnetwork_analysis/FSLNets;
addpath(sprintf('%s/usr/local/fsl/6.0.3/etc/matlab',getenv('fsl')));
addpath /PATH/TO/FSLnetwork_analysis/L1precision;
ts_dir = 'Network1.dr';
ts = nets_load(ts_dir, 1.5, 0);
group_maps = 'Network1';
ts.DD = [1:14];
N1_Pridgep_nets = nets_netmats(ts,1,'ridgep',0.1);
[N1_Znet_P,N1_Mnet_P]=nets_groupmean(N1_Pridgep_nets,1);
[p_uncorrected,p_corrected]=nets_glm(N1_Pridgep_nets,'design.mat','design.con',1);
nets_edgepics(ts,group_maps,N1_Znet_P,reshape(p_corrected(1,:),ts.Nnodes,ts.Nnodes),2);
nets_edgepics(ts,group_maps,N1_Znet_P,reshape(p_corrected(2,:),ts.Nnodes,ts.Nnodes),2);
nets_boxplots(ts,N1_Pridgep_nets,13,1,-1);
nets_boxplots(ts,N1_Pridgep_nets,12,10,-1);

#extract strength value for the first edge
IC1=13;
IC2=1;
i=(IC1-1)*ts.Nnodes + IC2;
Ngroup1=ts.Nsubjects/2;
Baseline_edge=N1_Pridgep_nets(1:Ngroup1,i);
Post-CRS_edge =N1_Pridgep_nets(Ngroup1+1:end,i);

#extract strength value for the second edge
IC1=12;
IC2=10;
i=(IC1-1)*ts.Nnodes + IC2;
Ngroup1=ts.Nsubjects/2;
Baseline_edge=N1_Pridgep_nets(1:Ngroup1,i);
Post-CRS_edge =N1_Pridgep_nets(Ngroup1+1:end,i);

## Same steps repeated for other networks and the control group
```
